# Relative Value Encoding in Large Language Models: A Multi-Task, Multi-Model Investigation

**DOI:** 10.1162/opmi_a_00209

**Published:** 2025-05-09

**Authors:** William M. Hayes, Nicolas Yax, Stefano Palminteri

**Affiliations:** Department of Psychology, Binghamton University, New York, NY, USA; Laboratoire de neurosciences cognitives et computationnelles, Institut national de la santé et de la recherche médicale, Paris, France; Département d’études cognitives, Ecole normale supérieure - PSL Research University, Paris, France; FLOWERS Lab, Institut national de recherche en informatique et en automatique, Bordeaux, France

**Keywords:** artificial intelligence, in-context learning, machine psychology, decision making, multi-armed bandits

## Abstract

In-context learning enables large language models (LLMs) to perform a variety of tasks, including solving reinforcement learning (RL) problems. Given their potential use as (autonomous) decision-making agents, it is important to understand how these models behave in RL tasks and the extent to which they are susceptible to biases. Motivated by the fact that, in humans, it has been widely documented that the value of a choice outcome depends on how it compares to other local outcomes, the present study focuses on whether similar value encoding biases apply to LLMs. Results from experiments with multiple bandit tasks and models show that LLMs exhibit behavioral signatures of relative value encoding. Adding explicit outcome comparisons to the prompt magnifies the bias, impairing the ability of LLMs to generalize from the outcomes presented in-context to new choice problems, similar to effects observed in humans. Computational cognitive modeling reveals that LLM behavior is well-described by a simple RL algorithm that incorporates relative values at the outcome encoding stage. Lastly, we present preliminary evidence that the observed biases are not limited to fine-tuned LLMs, and that relative value processing is detectable in the final hidden layer activations of a raw, pretrained model. These findings have important implications for the use of LLMs in decision-making applications.

## INTRODUCTION

In recent years, large language models (LLMs) have captured the attention of the media, industry, and the scientific community (Fowler, 2024; Newport, 2023; Ornes, 2024). LLMs are massive neural networks, based on the transformer architecture (Vaswani et al., 2017), that are trained on a vast amount of text data to predict the next token in a sequence. Yet, even with this rather simple training objective, LLMs possess many emergent abilities that extend beyond next-token prediction (Wei, Tay, et al., 2022). For example, LLMs can translate from one language to another, answer questions, unscramble words, classify text, solve math problems, and perform many other language-based tasks at a high level.

These tasks are accomplished through in-context learning (Brown et al., 2020): using only the contextual information contained in the prompt to accomplish a novel task, without any gradient updates or fine-tuning. In-context learning gives LLMs the human-like ability to perform new tasks from just a few examples (few-shot learning) or from instructions alone (zero-shot learning) (Kojima et al., 2022). Larger LLMs with hundreds of billions or even trillions of parameters are particularly good at in-context learning, especially as the number of examples provided in the prompt increases (Brown et al., 2020; Wei, Wang, et al., 2022).

Due to their impressive in-context learning abilities, LLMs are being increasingly applied to solve real-world problems (Liang et al., 2023; Sha et al., 2023; Thirunavukarasu et al., 2023). Of particular interest, LLMs are now being used to augment reinforcement learning (RL) algorithms (Cao et al., 2024). Reinforcement learning encompasses sequential decision problems in which agents interact with the environment through trial-and-error feedback to maximize reward (Sutton & Barto, 2018). Within the RL framework, LLMs can play many different roles. They may assist in processing information from the environment or make decisions based on the observations and instructions provided (Cao et al., 2024). Given the applications of LLMs in RL and their potential to function as autonomous agents (Park et al., 2023; Wang et al., 2024; Xi et al., 2023), it is important to understand how LLMs behave in sequential decision problems that require learning to maximize reward (Demircan et al., 2024; Krishnamurthy et al., 2024; Schubert et al., 2024).

One promising approach for understanding the behavioral tendencies of LLMs in RL and other kinds of tasks is to treat the models as participants in psychological experiments (Hagendorff et al., 2023). This “machine psychology” approach has already proven successful at revealing similarities and differences between human and LLM decision making in multiple domains (Aher et al., 2023; Binz & Schulz, 2023; Chen et al., 2023; Coda-Forno et al., 2023, 2024; Horton, 2023; Schubert et al., 2024; Yax et al., 2024). Unlike traditional methods for evaluating LLMs, which rely on overall performance benchmarks across a multitude of tasks (Hendrycks et al., 2020; Srivastava et al., 2022; Zheng et al., 2023), machine psychology tries to characterize the specific behavioral patterns exhibited by LLMs using a combination of carefully designed experiments and computational cognitive modeling (Coda-Forno et al., 2024; Schubert et al., 2024).

**Figure 1.**
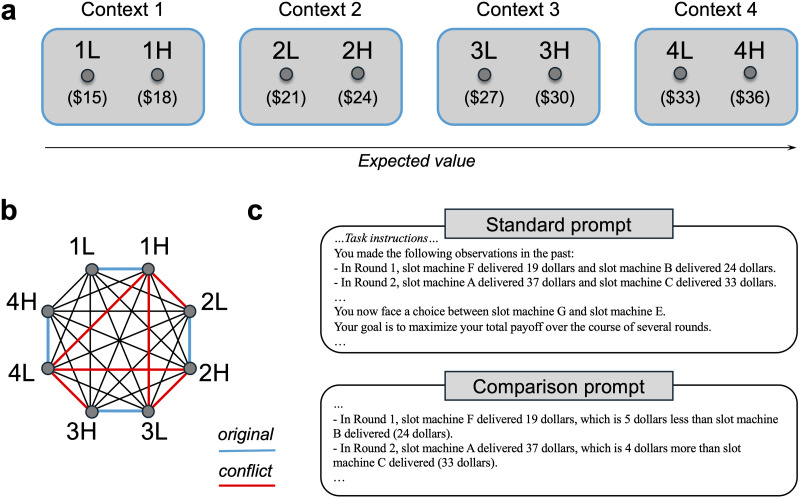
(a) A bandit task with eight options grouped into four learning contexts. Each learning context has a lower value option and a higher value option. During the initial learning phase, the options produce Gaussian-distributed rewards (means in parentheses, standard deviation of $1). (b) All possible pairs of options in the transfer test. Blue lines show the originally learned pairs. Red lines show pairs for which absolute and relative values conflict, such that learned relative values favor the option with lower absolute value. (c) Prompt designs used in all experiments. The comparison prompt added explicit comparisons between the local outcomes delivered in each round.

Here, we leverage the machine psychology approach to investigate the ability of LLMs to learn from past outcomes presented in-context to maximize reward in RL tasks. The focus is on relatively simple bandit tasks because of their simplicity and tractability, and because past studies have successfully used them to shed light on fundamental properties of reward encoding and belief updating (Lefebvre et al., 2017; Palminteri, 2025; Palminteri et al., 2015, 2017). In our bandit tasks, options are grouped in fixed pairs (or triplets) during the initial “learning” phase. Figure 1a shows an example task with four pairs of options (i.e., “contexts”), adapted from a study by Hayes and Wedell (2023a). Each learning context has a lower value option and a higher value option with Gaussian-distributed rewards. The goal is to make choices that maximize payoffs, which means that agents should learn to exploit the higher value option in each context. People do this very efficiently with trial-by-trial feedback from both options (i.e., complete feedback; (Hayes & Wedell, 2023a).

Analyzing human behavior in this kind of bandit task has revealed that choice outcomes are encoded in a relative or context-dependent fashion (Palminteri & Lebreton, 2021). Relative outcome encoding causes the learned value of an option to be heavily biased by how its outcomes compare to the outcomes of the other option in the same learning context. This can be experimentally demonstrated by including a transfer test after the learning phase, consisting of choices between all possible pairs of options (Figure 1b). The objective is still to maximize payoffs, but feedback is no longer provided. If people learned the absolute (context-independent) values of each option, they should be highly accurate at making reward-maximizing choices. On the other hand, if they only learned the relative (context-dependent) values, they should make correct choices whenever relative values favor the maximizing option (the blue lines in Figure 1b), and incorrect choices whenever relative values favor the non-maximizing option (red lines in Figure 1b). Consistent with relative value learning, people frequently fail to select maximizing options in the transfer test when those options were paired with a better option during the learning phase (Hayes & Wedell, 2023a). Thus, relative encoding can undermine the ability to generalize learned option values to new contexts or situations (Bavard et al., 2018; Klein et al., 2017).

The present study examines relative outcome encoding in LLMs using bandit tasks adapted from human studies and translated into natural language. While there is some existing evidence for relative value biases in LLMs, it is currently limited to a single task and just two models (Hayes et al., 2024). Here, we present results from a multi-task, multi-model investigation that encompassed five different bandit tasks and four LLMs. Two different prompt designs were examined, one in which the outcomes from the chosen and unchosen options in each round are listed in a neutral fashion (standard prompt), and another that incorporates explicit relative comparisons between the outcomes (comparison prompt; Figure 1c). Adding explicit comparisons between the outcomes in each round should make it easier to differentiate between options that belong to the same learning context, since these options (and their outcomes) are always paired together in the prompt. In humans, factors that facilitate direct comparisons between the options in each context tend to improve learning phase performance while simultaneously enhancing relative value processing, which can impair transfer performance (Bavard et al., 2021). Based on this finding and preliminary evidence from a prior study (Hayes et al., 2024), it is predicted that relative value effects would be observed in both prompt conditions but to a greater degree in the comparison condition.

To foreshadow the results, all four LLMs generally learned to select reward-maximizing options with greater-than-chance accuracy. While transfer test performance was also above chance overall, behavioral signatures of relative value learning were found in four out of the five bandit tasks, especially using the comparison prompt (Figure 1c). The comparison prompt generally improved learning phase performance while having a detrimental effect on transfer performance. This mirrors the trade-off observed in humans, where factors that facilitate relative processing make it easier to learn which options are better in the original learning contexts but undermine the ability to generalize learned option values to new contexts (Bavard et al., 2021). Additionally, using computational cognitive modeling, we find that LLM behavior in our bandit tasks is well-described by simple RL algorithms that encode a combination of relative and absolute outcomes. Taken together, these findings have important implications for the study of in-context reinforcement learning in LLMs, highlighting the prevalence of human-like relative value biases.

## METHODS

### Models

Four LLMs were tested in the main experiments. All four are based on the transformer architecture (decoder-only), pretrained on next-token prediction, and fine-tuned for chat or instruction following. Two proprietary models, gpt-3.5-turbo-0125 and gpt-4-0125-preview (OpenAI et al., 2023), were accessed via the OpenAI API. The other two models, llama-2-70b-chat (Touvron et al., 2023) and mixtral-8x7b-instruct (Jiang et al., 2024), are open-source and were accessed via the Hugging Face Inference API. The experiments reported in the Results were run in March and April 2024. The models’ temperature parameter was set to zero to obtain deterministic responses (Coda-Forno et al., 2024; Schubert et al., 2024). All other parameters were kept at their default values.

### Design

Five bandit tasks were selected from prior studies (Bavard et al., 2018; Bavard & Palminteri, 2023; Hayes & Wedell, 2023a, 2023b; Vandendriessche et al., 2023) that varied on a range of structural features. Table 1 summarizes the major differences between them (see Figure S1 for a detailed summary). Henceforth, the abbreviations in Table 1 will be used to refer to the tasks.

**Table 1.** Summary of the bandit tasks

Feature	Task #1 (B2018)	Task #2 (V2023)	Task #3 (HW2023a)	Task #4 (BP2023)	Task #5 (HW2023b)
Options	8	4	8	10	8
Learning contexts	4 binary	2 binary	4 binary	2 binary, 2 ternary	4 binary
Reward distribution	Bernoulli	Bernoulli	Gaussian	Gaussian	Bernoulli
Reward range	[−1, 1]	[0, 1]	∼[12, 39]	∼[8, 92]	[10, 44]
Reward currency	euros	points	dollars	dollars	dollars
Learning trials^a^	48	60	60	60	60
Transfer trials	28	12	28	45	28

*Note*. Tasks were adapted from the following studies: Bavard et al. (2018) (B2018), Vandendriessche et al. (2023) (V2023), Hayes and Wedell (2023a) (HW2023a), Bavard and Palminteri (2023) (BP2023), Hayes and Wedell (2023b) (HW2023b).

^a^ The total number of learning trials are divided evenly among the learning contexts.

At a broad level, four to ten options were grouped together in fixed learning contexts. The learning contexts were mostly binary (comprised of two options each), but one task (BP2023) included two ternary contexts. The options produced probabilistic rewards from either a Bernoulli or Gaussian distribution. Two of the learning contexts in the B2018 task included losses. For the tasks with Bernoulli-distributed outcomes, the relative frequencies were made to match the underlying probabilities exactly in the simulations.

All tasks consisted of a learning phase and a transfer test. Each learning context (i.e., option grouping) was presented 12–30 times during the learning phase for a total of 48–60 trials, randomly interleaved. The transfer test consisted of one or two (V2023 only) choices for all possible pairs of options.

### Procedure

The experimental procedures were based on prior studies of RL behavior in LLMs (Binz & Schulz, 2023; Coda-Forno et al., 2024; Hayes et al., 2024; Schubert et al., 2024). All instructions, choice stimuli, and rewards were translated to natural language. The options were described as “slot machines,” with the letters A-J randomly assigned at the beginning of each simulation run depending on the number of options in the task. The instructions varied slightly across tasks, but the gist was always that the agent would be making several choices with the goal of maximizing payoffs (see Figure S2 for task-specific prompts).

In each round the task, the end of the prompt stated, “You now face a choice between slot machine [X] and slot machine [Y]” with X and Y replaced by the letters that were assigned to the currently available options. The order in which the options were listed was randomized on a trial-by-trial basis. After restating the goal to maximize payoffs, the last sentence specified the desired response format: “Which slot machine do you choose? Give your answer like this: I would choose slot machine _. Do not explain why.” This format was chosen to facilitate data analysis and avoid lengthy responses. After the LLM responded with the chosen slot machine, if the current trial was part of the learning phase, outcomes from the chosen and unchosen option(s) were drawn from the corresponding distributions and appended to the history of previous outcomes (complete feedback). The updated outcome history was then used in the prompt for the next trial. If the current trial was part of the transfer test, the outcome history was left unchanged (no feedback). Two prompt designs were tested. In the standard prompt, the outcomes for both options in each round were listed in a neutral fashion. In the comparison prompt, explicit comparisons between the outcomes were added (see Figure 1c). All other parts of the prompt were the same in both conditions.

To reduce any dependence on specific outcome sequences, 30 independent experiments with randomly sampled outcomes were run for each combination of task (5 levels), LLM (4 levels), and prompt condition (2 levels), for a total of 1200 runs. All experiments were carried out on a CPU.

## RESULTS

### Choice Accuracy

The explicit goal in each bandit task was to maximize payoffs. Choice accuracy, defined as the proportion of reward-maximizing choices, was computed for each experiment run and analyzed using a 5 (Task) × 4 (LLM) × 2 (Prompt) between-subjects analysis of variance (ANOVA).^1^ Means and 95% confidence intervals are reported for key comparisons. The learning phase and transfer test were analyzed separately.

**Figure 2.**
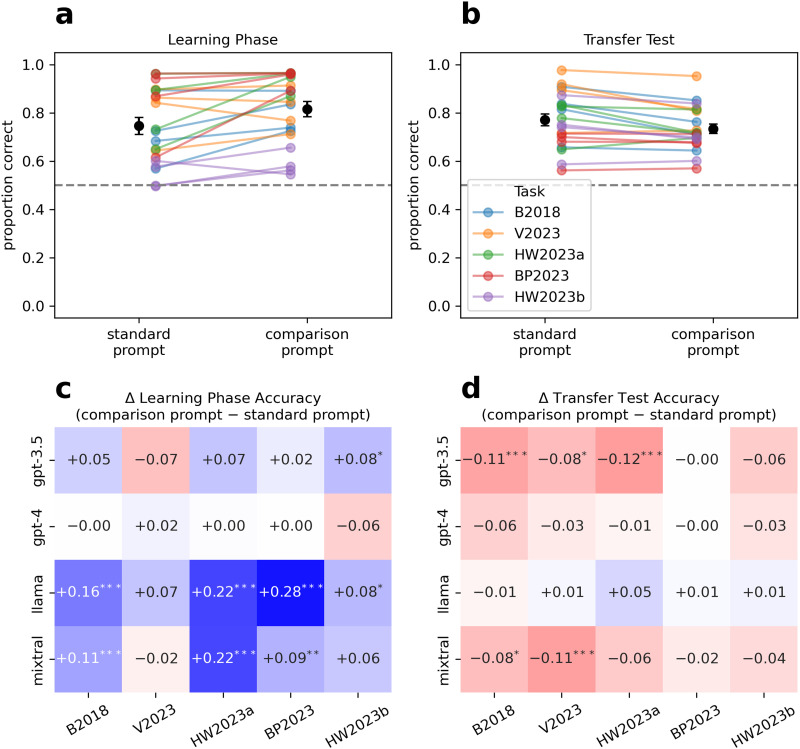
(a–b) Mean choice accuracy (proportion of reward-maximizing choices) in the learning phase and transfer test. Each colored point represents the mean accuracy for a specific combination of bandit task, model, and prompt condition across 30 runs (lines connect the same model-task combination). Means and standard errors are also shown. (c–d) Pairwise contrasts for the effect of prompt design, broken down by task and model. *p < .05; **p < .01; ***p < .001 (Bonferroni-adjusted for 20 tests).

Learning phase accuracy was generally well above chance, indicating that LLMs were successful at learning which options were better in each local context from the information contained in the prompt (Figure 2a). Collapsing across bandit tasks and models, learning phase accuracy was significantly higher using the comparison prompt (*M* = .815, 95% CI [.807, .823]) compared to the standard prompt (*M* = .746, [.738, .754]), *F*(1, 1160) = 147.39, *p* < .001, *η*_*p*_^2^ = 0.11. All other main effects and interactions were also significant (*p*s < .001; see Table S1 for full ANOVA results). Of note, learning phase accuracy tended to be higher for the GPT models, especially gpt-4-0125-preview, and for the bandit tasks with Gaussian reward distributions (HW2023a, BP2023).

Transfer test accuracy was also above chance on average (Figure 2b), indicating that LLMs were mostly successful at generalizing learned option values to unseen choice sets.^2^ However, in contrast to the learning phase, transfer accuracy was significantly lower, on average, using the comparison prompt (*M* = .734, 95% CI [.727, .741]) compared to the standard prompt (*M* = .771, [.764, .778]), *F*(1, 1160) = 53.52, *p* < .001, *η*_*p*_^2^ = 0.04. The other main effects and two-way interactions were significant (*p*s < .005), but the three-way interaction was not (see Table S2). Collapsing across bandit tasks and prompt conditions, the highest transfer accuracy was achieved by gpt-4-0125-preview (*M* = .847, 95% CI [.837, .857]), and the lowest by llama-2-70b-chat (*M* = .641, [.631, .651]). Transfer accuracy also varied across bandit tasks when collapsing across models and prompt conditions, being highest in the V2023 task (*M* = .852, [.841, .863]) and lowest in the BP2023 task (*M* = .662, [.651, .673]).

The bottom panels of Figure 2 show the effect of prompt design for all model-task combinations. In the learning phase, the comparison prompt was most beneficial for the two open-source models, especially in the tasks with Gaussian reward distributions (Figure 2c). In contrast, gpt-4-0125-preview, which performed at or near ceiling by the end of the learning phase in four of the five tasks, did not benefit from explicit comparisons in the prompt. The pattern was considerably different for the transfer test, where the comparison prompt had a mostly negative effect (Figure 2d). The one exception was llama-2-70b-chat, for which explicit comparisons led to a slight overall improvement in transfer accuracy.

### Relative Value Bias

To analyze relative value bias, one needs to define “relative value” in a way that works for all five bandit tasks. The relative value of an option could depend on whether it was the optimal choice (based on expected value) in its original learning context. However, people sometimes prefer sub-optimal options if they give better outcomes *most of the time* (Hayes & Wedell, 2023b). Strikingly, LLMs also exhibited this tendency in the HW2023b task (see Figure S13). Thus, we chose to define the relative value of an option based on how *most* of its outcomes compared to the outcomes of the other option(s) in its original learning context. Options that frequently gave better relative outcomes have higher relative value, and options that frequently gave worse relative outcomes have lower relative value.

Relative value bias was analyzed using the models’ choices in the transfer test. On congruent trials, the option with higher relative value from the learning phase was the correct (maximizing) choice, but on incongruent trials, the option with higher relative value from the learning phase was the incorrect (non-maximizing) choice (see Figure 1b for an example).^3^ Using this information, one can easily calculate the rate at which an ideal, reward-maximizing agent would choose options with higher relative value in the transfer test. This ideal choice rate differed across the five bandit tasks, ranging from .50 in HW2023b to .969 in BP2023. Relative value bias was said to occur when an LLM’s empirical choice rate exceeded the ideal. Because an ideal agent would always choose the higher relative value option on congruent trials, but never on incongruent trials, relative value bias can only occur if an LLM occasionally picks the “wrong” option on incongruent trials.

LLMs frequently chose options with higher relative value in excess of an ideal agent, mainly when using the comparison prompt, and most noticeably in the B2018, HW2023a, and HW2023b tasks (see Figure S3). A Task × LLM × Prompt ANOVA confirmed that options with higher relative value were chosen at a higher rate with the comparison prompt (*M* = .829, 95% CI [.820, .838]) than with the standard prompt (*M* = .698, [.689, .707]), *F*(1, 1159) = 440.61, *p* < .001, *η*_*p*_^2^ = 0.28, averaging across bandit tasks and models. All other main effects and interactions were significant as well (*p*s < .001) (see Table S3). Table 2 shows the estimated marginal mean choice rates of options with higher relative value as a function of prompt condition and task, collapsing across LLMs. When compared to an ideal agent, the comparison prompt led to significant relative value bias in four of the five tasks, while the standard prompt led to significant relative bias in just two tasks.

**Table 2.** Estimated mean choice rates for options with higher relative value in the transfer test

Prompt	Task	Ideal	Mean	95% CI	Bias
Standard	B2018	0.625	0.643	[0.623, 0.662]	
	V2023	0.750	0.738	[0.718, 0.757]	
	HW2023a	0.625	0.721	[0.701, 0.740]	✓
	BP2023	0.969	0.811	[0.792, 0.831]	
	HW2023b	0.500	0.577	[0.558, 0.597]	✓
Comparison	B2018	0.625	0.776	[0.757, 0.795]	✓
	V2023	0.750	0.787	[0.768, 0.807]	✓
	HW2023a	0.625	0.926	[0.906, 0.945]	✓
	BP2023	0.969	0.936	[0.916, 0.955]	
	HW2023b	0.500	0.721	[0.701, 0.740]	✓

*Note*. Results are collapsed across LLMs. Bias column indicates whether there was a significant relative value bias (if the 95% CI for the mean choice rate was above the ideal agent).

Figures S4–S7 in the Supplemental Material show the pairwise choice patterns in the transfer test for all tasks except BP2023, which did not include incongruent trials. Violations of expected value (EV) maximization on incongruent transfer trials were more frequent for choices between options with similar EVs. However, they also occurred for choices between options with larger EV differences, especially using the comparison prompt. For example, in the HW2023a task, when the comparison prompt was used, gpt-3.5-turbo-0125, llama-2-70b-chat, and mixtral-8x7b-instruct all exhibited a strong preference for option 1H (EV = $18) over option 4 L (EV = $33), despite the large gap in expected payoffs.

### Cognitive Modeling

Computational cognitive models were used to provide an interpretable, mechanistic account of LLM in-context learning in our bandit tasks. The models were simple RL algorithms with three basic components: (1) a subjective value function, which determines how rewards are encoded, (2) a learning function, which determines how expectancies are updated in response to feedback, and (3) a response function, which maps expectancies onto actions.

The general form of the subjective value function is as follows:

$$v\left(x_{i,t}\right)=\left(1-\omega \right)\cdot x_{i,t}^{ABS}+\omega \cdot x_{i,t}^{REL}$$ (1)

where *x*_*i*,*t*_ is the *i*th reward on trial *t*, $x_{i,t}^{ABS}$ is the value of *x*_*i*,*t*_ normalized by the full range of rewards experienced so far across all learning contexts,^4^ $x_{i,t}^{REL}$ is the value of *x*_*i*,*t*_ normalized by the range of rewards *on the current trial only*, and *ω* is the relative encoding parameter. Similar weighted encoding schemes are frequently used to model human RL behavior (Bavard et al., 2018; Hayes & Wedell, 2021, 2023b; Molinaro & Collins, 2023). Note that if *ω* = 0, subjective values are proportional to the absolute reward magnitudes and the model predicts no effects of the local context. We refer to this special case as the ABS model. On the other hand, if *ω* = 1, the subjective value of an outcome is entirely determined by how it compares to the other local outcome(s) experienced on that trial. We refer to the more general, hybrid model with *ω* freely estimated as the REL model.

Once subjective values are formed, learning is modeled using a basic prediction error-driven updating function (Rescorla & Wagner, 1972):

$$Q_{t+1}\left(a_{i}\right)=Q_{t}\left(a_{i}\right)+\alpha \cdot \left(v\left(x_{i,t}\right)-Q_{t}\left(a_{i}\right)\right)$$ (2)

where *Q*_*t*+1_(*a*_*i*_) is the updated expectancy for option *a*_*i*_, *Q*_*t*_(*a*_*i*_) is the prior expectancy, and *α* is the learning rate parameter. In bandit tasks, LLMs seem to update expectancies more in response to feedback that confirms prior beliefs and discount feedback that contradicts them (Schubert et al., 2024). This propensity has also been observed in humans and is thought to represent a kind of confirmation bias (Palminteri et al., 2017). Based on that work, we include models with separate learning rates for confirmatory outcomes (*α*_*CON*_) and disconfirmatory outcomes (*α*_*DIS*_).^5^

Learned expectancies are mapped to choice probabilities using the softmax function with an inverse temperature parameter *β*:

$$p\left(a_{i}\right)=\frac{\exp\left(\beta \cdot Q_{t}\left(a_{i}\right)+b\cdot \delta \left(a_{i}\right)\right)}{\sum_{k}\exp\left(\beta \cdot Q_{t}\left(a_{k}\right)+b\cdot \delta \left(a_{k}\right)\right)}$$ (3)

where *δ*(·) is an indicator function that equals 1 if its argument is the first option listed in the prompt. Positive values of *b* increase preference for the first-listed option, regardless of its value. Thus, choice probabilities depend on both learned expectancies and, potentially, a form of response bias. Finally, some models used separate inverse temperatures (*β*) for the learning phase and transfer test.

The factorial combination of absolute or (partially) relative encoding, one or two learning rates, and one or two inverse temperatures results in eight candidate models. Models were fit to the choice data using maximum likelihood methods. A single set of parameters was estimated for each combination of LLM, task, and prompt condition, each time pooling the data across the 30 experiment runs, with *Q* values reset to 0.5 on the first trial of each run. Models were compared using the Bayesian information criterion (BIC), where models with lower BIC are preferred. BIC tends to favor simpler models with fewer parameters, and this trend was also observed in model recovery simulations. However, recovery of the correct, data-generating model was well above chance across the five bandit tasks (average recovery rate: 66% [chance = 12.5%]). When the wrong model was selected, it was almost always a simpler model that possessed the correct subjective value function, indicating that absolute and relative valuation are at least highly distinguishable in these bandit tasks. Full model recovery results are presented in the Supplemental Material (Figure S8).

As a robustness check, we performed an additional model comparison using a leave-one-out cross-validation procedure. For each combination of LLM, task, and prompt condition, we fit the pooled data from 29 of the 30 runs and computed the log-likelihood of the data from the remaining run.^6^ This procedure was repeated for each of the runs, resulting in 30 out-of-sample log-likelihood values. To make these log-likelihoods more interpretable, we converted them to a pseudo-*R*^2^ metric using the formula 1 − *LL*_*M*_/*LL*_0_, where *LL*_*M*_ is the (out-of-sample) log-likelihood for the model in question and *LL*_0_ is the (out-of-sample) log-likelihood for a null model that predicts chance probabilities on every trial (.50 for binary choice, .33 for ternary choice). The pseudo-*R*^2^ values were then averaged across runs. The model comparison results were very similar using this metric, and thus we focus on BIC comparisons here and report the cross-validation results in the Supplemental Material (Table S5).

**Figure 3.**
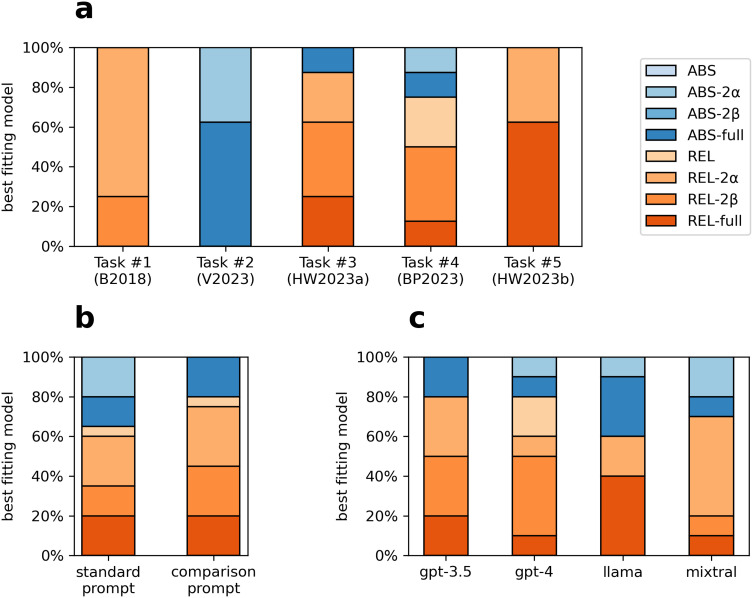
Best-fitting cognitive models (%) across tasks (a), prompt designs (b), and LLMs (c) according to BIC. Models with hybrid (absolute and relative) outcome encoding (REL) generally outperformed absolute encoding models (ABS), and models that used separate learning rates and/or separate inverse temperatures generally outperformed models with a single learning rate or inverse temperature.

Figure 3 shows the best-fitting models based on BIC across bandit tasks, prompt conditions, and LLMs (see Table S4 for the BIC values). With the exception of the V2023 task, models that incorporated relative encoding were more frequently selected as the best description of LLM behavior. Models that used separate learning rates and/or separate inverse temperatures were more frequently selected than the models with a single learning rate or inverse temperature.

Figure S3 in the Supplemental Material shows the fit of the ABS-full and REL-full models to the data. The ABS-full model was able to capture some of the relative value biases even though it assumes unbiased outcome encoding. This was likely due to the use of separate learning rates: If *a*_*CON*_ > *a*_*DIS*_, the models tend to repeat previous choices, which would generally favor options with higher relative value. However, it was clearly not able to capture the magnitude of the biases as well as the REL-full model. See Figures S9–S13 for plots showing the fit of the REL-full model to the learning and transfer patterns in each task.

**Figure 4.**
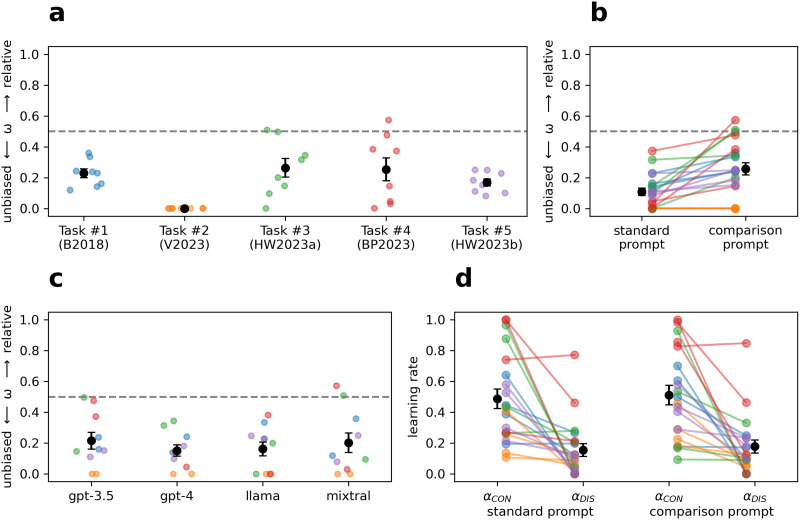
(a) Estimated relative encoding parameters from the REL-full model across tasks, (b) prompt conditions, and (c) LLMs. (d) Estimated learning rates. In each panel, black points show the means and standard errors. The estimated relative encoding parameters were mostly above zero except for the V2023 task, higher when using the comparison prompt, and similar across LLMs. Estimated learning rates were generally higher for confirmatory outcomes compared to disconfirmatory outcomes, consistent with confirmation bias in belief updating.

The parameters of the REL-full model were examined next. The mean estimates for the relative encoding parameter (*ω*) were consistently above 0 for all tasks except V2023 (Figure 4a). In line with the model-free results, *ω* estimates were significantly higher with the comparison prompt (*M* = .257, 95% CI [.173, .340]) than with the standard prompt (*M* = .109, [.058, .160]), *t*(19) = 4.16, *p* < .001, *d* = 0.93 (paired *t*-test; Figure 4b). All four LLMs exhibited similar degrees of relative encoding on average (Figure 4c). Finally, consistent with prior work (Schubert et al., 2024), the estimates for *α*_*CON*_ were higher than the estimates for *α*_*DIS*_ using both the standard prompt (.488 ± .137 vs. .155 ± .088) and the comparison prompt (.512 ± .137 vs. .178 ± .092) (Figure 4d).

Parameters that control the mapping from expectancies to choice probabilities were also examined. The average inverse temperature (*β*) was higher in the learning phase (*M* = 17.934, 95% CI [10.490, 25.378]) than in the transfer test (*M* = 11.128, [7.483, 14.773]), which may be interpreted as a kind of “forgetting,” and the average position bias (*b*) was significantly positive (*M* = 1.178, [1.030, 1.326]), indicating a bias toward choosing the first option listed. See Figure S14 for a comparison of fitted parameters across LLMs.

### Analysis of Hidden States

**Figure 5.**
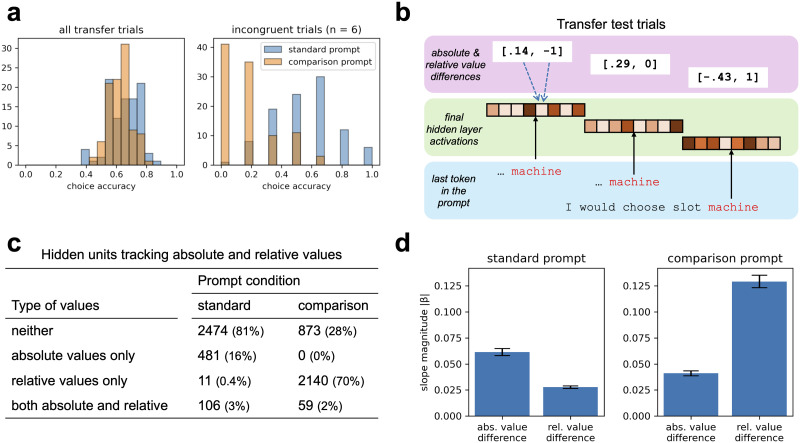
Analysis of hidden states using gemma-7b. The model performed a version of the HW2023a task with 40 learning trials and a transfer test (*n* = 100 runs per prompt condition). (a) Distributions of choice accuracy (proportion of reward-maximizing choices) across all transfer trials (left) and restricted to the six incongruent trials in which relative values favored the non-maximizing option (right). (b) On each transfer trial, activations for the last token in the prompt were recorded from the model’s final hidden layer. Linear regression was used to predict the trial-to-trial activations in each hidden unit from the trial-to-trial differences in absolute and relative values for the available choice options, both normalized between 0 and 1. Each hidden unit was modeled separately for a total of 3072 regressions. (c) The number of hidden units with significant partial regression coefficients for absolute value differences, relative value differences, or both, corrected for multiple comparisons (see Supplemental Material). (d) Average unsigned partial regression coefficients. Absolute value differences had a stronger average effect on gemma-7b’s hidden layer activations than relative value differences using the standard prompt. The reverse was true using the comparison prompt. Means and standard errors calculated across 3072 hidden units.

The four LLMs in the main experiments were all fine-tuned for chat or instruction following. In an additional set of experiments, we ran gemma-7b (Gemma Team & Google DeepMind, 2024), a pretrained model with no fine-tuning, through the HW2023a task and found that it too showed robust relative value biases. In the transfer test, gemma-7b selected options with higher relative value 61% of the time using the standard prompt, but 91% of the time using the comparison prompt (ideal agent: 62.5%). This led to a striking accuracy difference on incongruent trials (Figure 5a).

The effect of the comparison prompt was also clearly reflected in the model’s hidden states. To demonstrate, we ran gemma-7b through the transfer test *n* = 100 times and extracted the final hidden layer activations for the last token in the prompt on each trial (Figure 5b; see the Supplemental Material for details). A linear regression model was then fit to the activations in each hidden unit separately (*n* = 3072 regressions). The two predictors were (1) the difference in absolute values and (2) the difference in relative values for the available options on each trial, both normalized between 0 and 1. Using a very conservative correction for multiple tests, the regression coefficient for relative values was significant for only 3% of the hidden units using the standard prompt, but 72% using the comparison prompt. In contrast, the regression coefficient for absolute values was significant for 19% of the hidden units with the standard prompt but just 2% with the comparison prompt (Figure 5c). Further, the average unsigned coefficient for relative values was greater than the average unsigned coefficient for absolute values using the comparison prompt, *t*(3071) = 23.83, *p* < .001, *d* = 0.43, but the opposite was true using the standard prompt, *t*(3071) = 16.35, *p* < .001, *d* = 0.30 (paired *t*-test) (Figure 5d).

These preliminary experiments with gemma-7b demonstrate that relative value biases are observable even in the absence of fine-tuning for chat or instruction following. Further, the model’s hidden layer activations appear to track task-relevant information on a trial-to-trial basis in a way that is highly dependent on the framing of the prompt. Incorporating explicit outcome comparisons in the prompt significantly enhances the representation of relative values in the model’s final hidden layer.

## DISCUSSION

The present research examined the in-context RL abilities of LLMs in simple bandit tasks (Binz & Schulz, 2023; Coda-Forno et al., 2024; Hayes et al., 2024; Krishnamurthy et al., 2024; Schubert et al., 2024). Our primary goal was to use methods from cognitive science, including carefully controlled experiments and computational modeling, to understand a specific decision-making bias in LLMs: “relative value bias.” Relative value bias refers to a learned preference for options that frequently gave better relative outcomes in one choice context, even when those same options are not the most rewarding in a new context. While this bias has been repeatedly observed in humans and certain animal species (Bavard et al., 2018, 2021; Hayes & Wedell, 2023a; Klein et al., 2017; Palminteri & Lebreton, 2021; Pompilio & Kacelnik, 2010; Solvi et al., 2022), it has only just begun to be studied in LLMs (Hayes et al., 2024). By translating bandit tasks from prior human studies into natural language, we were able to devise an experimental paradigm for examining relative value bias in LLMs. We found consistent evidence for the bias across five different tasks and four models (gpt-3.5-turbo-0125, gpt-4-0125-preview, llama-2-70b-chat, and mixtral-8x7b-instruct). We showed that the bias can be enhanced by adding explicit relative comparisons between outcomes to the prompt. Lastly, we demonstrated that relative values are represented in the final hidden layer activations of a raw, pre-trained LLM (gemma-7b), and to a much greater extent when the prompt contains explicit outcome comparisons.

While other decision-making biases and reasoning errors have been documented in LLMs (e.g., Binz & Schulz, 2023; Echterhoff et al., 2024; Schubert et al., 2024; Yax et al., 2024), we want to emphasize just how remarkable it is that highly advanced LLMs exhibit relative value bias. In all of our experiments, a full history of previous outcomes from all options is available in the prompt. On every trial, a decision-making agent could easily figure out which option is the reward-maximizing choice by simply summing or averaging the rewards from past trials. This is quite different from studies with humans, where the outcomes from previous trials must be stored in memory. Yet, despite all of the necessary information being immediately available in the prompt, LLMs frequently chose an option with a lower average reward over an option with a higher average reward, simply because the former was repeatedly paired with a worse option and the latter with a better option. Importantly, gpt-4-0125-preview, the most advanced LLM of the models we tested, exhibited the strongest degree of relative value bias overall. This suggests that relative value bias is not something that appears only in smaller LLMs but goes away in larger LLMs. In fact, our results suggest it may even get worse (up to a point) with increasing model capacity.

We demonstrated that the choice behavior of LLMs was generally best explained by RL models that encode a combination of absolute and relative outcome values (Bavard et al., 2018; Hayes & Wedell, 2021). However, for one of the five bandit tasks that we tested (V2023; Vandendriessche et al., 2023), models with absolute outcome encoding were preferred (Figure 3). One difference between the V2023 task and the others is that it uses only two learning contexts (i.e., fixed option pairings) instead of four. However, we do not believe that the smaller number of contexts is the reason why absolute valuation models were preferred, because when we ran gpt-4-0125-preview (the most advanced LLM) in a two-context version of the HW2023a task (Hayes & Wedell, 2023a), we still observed a strong relative value bias. In fact, when gpt-4-0125-preview encountered a choice between options 1H (EV = $18) and 2 L (EV = $21) in the transfer test (an incongruent trial), it chose the maximizing option only once in 57 experiments.^7^ This preliminary evidence suggests that LLMs can exhibit relative value bias even in simpler bandit tasks with two learning contexts.

Instead, the specific outcome distributions used in the V2023 task may not be ideal for distinguishing between absolute and relative valuation. All four options gave the same binary outcomes (0 or 1) with different probabilities of reward: {0.1, 0.4} for the options in Context 1, {0.6, 0.9} for the options in Context 2. Using a prediction error-driven learning rule (Equation 2), if all outcomes are weighted equally, the learned *Q* values will gradually approximate the underlying expected values of the four options. However, if the rewarding outcomes (the 1’s) receive greater weight for option 1H, but the non-rewarding outcomes (the 0’s) receive greater weight for option 2 L, then the learned *Q* value for the former can surpass the learned *Q* value for the latter. But this is precisely what happens in the models that employ separate learning rates for confirmatory (*α*_*CON*_) and disconfirmatory (*a*_*DIS*_) outcomes: If *a*_*CON*_ > *a*_*DIS*_ (confirmation bias), outcomes that favor the agent’s current choice preferences (i.e., the 1’s for option 1H and the 0’s for option 2 L) are weighted more than the other outcomes. As a result, a bias toward higher relative value options can arise in the V2023 task simply due to confirmation bias, without the need to assume relative outcome encoding. In contrast, relative outcome encoding is needed to explain context-dependent choice biases in the other bandit tasks.

A limitation of the current work is its focus on models that have been fine-tuned for chat or instruction following using techniques such as reinforcement learning from human feedback (Christiano et al., 2017) or direct preference optimization (Rafailov et al., 2023). However, we presented preliminary evidence that relative value biases can appear even in the absence of fine-tuning and human feedback. Another limitation is that we did not consider prompting strategies that may help to mitigate relative value bias. In prior work, instructing the models in the prompt to estimate expected payoffs before making a choice all but eliminated the bias (Hayes et al., 2024). Other strategies, such as zero-shot chain of thought prompting (Kojima et al., 2022), could prove effective. Future work should continue searching for prompting strategies that can mitigate decision-making biases while extending this line of research to a broader set of LLMs and task designs (Coda-Forno et al., 2024).

Our results suggest that if an LLM is to be deployed in a real-world decision-making application, particularly one that requires (in-context) learning from past outcomes, its choices will likely be biased in favor of options with higher relative reward value, sometimes at the expense of absolute reward value. Further, our study demonstrates that subtle changes to the way that past outcomes are presented in the prompt can have dramatic effects on the ability of LLMs to generalize learned values to new situations. If each option belongs to a single choice set and the goal is simply to maximize payoffs within each set (a typical bandit task design), adding explicit local outcome comparisons to the prompt may facilitate in-context learning. However, if the same option can appear in more than one choice set, adding comparisons to the prompt can impair the ability to learn value representations that generalize across contexts.

To demonstrate these effects, we designed the bandit tasks so that the options that were relatively better in the learning phase are no longer optimal in the transfer test. Further, we withheld feedback in the transfer test so that the models would not update their learned representations. The same setup is used in human studies (Palminteri & Lebreton, 2021). These kinds of artificial conditions may not occur often in the real world, whether for human or LLM agents, so it remains unclear how often we should expect relative value bias to lead to negative consequences in real-world settings. Nevertheless, we believe that demonstrating relative value bias in LLMs is important on its own, so that scientists and engineers are aware of its existence and the factors that moderate it when designing applications.

Although we focused on a particular RL bias in LLMs, it should be emphasized that the models generally performed above chance in our bandit tasks when looking at overall maximization accuracy (Figure 1a, b). The observation that LLMs, despite being biased, are rather proficient reinforcement learners may come as a surprise if one focuses on the fact that reward maximization is not something these neural networks were explicitly trained to do (unlike deep Q-networks, whose training objective function is directly linked to reward; Li, 2017). Since the objective function in large language models is to maximize the probability of predicting the next token, their reinforcement learning abilities must therefore be considered emergent abilities (Lu et al., 2023). The presented results illustrate the principle that a multiplicity of complex proximal cognitive abilities (such as in-context reinforcement learning, incorporating human cognitive biases) can emerge as a byproduct of solving relatively simple distal goals (i.e., next-token prediction) (Hussain et al., 2025).

## ACKNOWLEDGMENT

The authors thank Can Demircan for helpful discussions regarding the analysis of hidden unit activations.

## FUNDING INFORMATION

SP is supported by the European Research Council under the European Union’s Horizon 2020 research and innovation program (ERC) (RaReMem: 101043804), and the Agence National de la Recherche (CogFinAgent: ANR-21-CE23-0002-02; RELATIVE: ANR-21-CE37-0008-01; RANGE: ANR-21-CE28-0024-01).

A portion of this work was presented at the 46th Annual Meeting of the Cognitive Science Society (CogSci 2024) and as a preprint: https://arxiv.org/abs/2405.11422.

## DATA AND CODE AVAILABILITY

Raw data and code can be found in the Github repository for this project: https://github.com/william-hayes/LLMs-biased-RL.

## Notes

^1^ Prior to analysis, *n* = 1401 invalid LLM responses that did not conform to the specified format were removed from the data (1.36% of all choice trials). Most of these (*n* = 1204) occurred in the BP2023 task.^2^ Choices between options with equal expected value were excluded from this analysis. These only occurred in the BP2023 task.^3^ Choices between options with tied relative values were excluded from this analysis.^4^ Using the range normalization formula: (*x* − *min*)/(*max* − *min*).^5^ Confirmatory outcomes are when the chosen option’s outcome is better than expected (positive prediction error) or the unchosen option’s outcome is worse than expected (negative prediction error). Disconfirmatory outcomes are the reverse.^6^ Because each run involved a different random sequence of outcomes, training and test data were independent.^7^ 60 total experiments were run with gpt-4-0125-preview, 30 with the standard prompt and 30 with the comparison prompt, in a modified version of the HW2023a task with two learning contexts: {1L, 1H} and {2L, 2H} (see Figure 1a). In three of the 60 experiments, the model made an invalid response when choosing between 1H and 2 L in the transfer test, resulting in 57 valid observations.

## Supplementary Material


