# Supplementary material for: Relative Value Encoding in Large Language Models: A Multi-Task, Multi-Model Investigation
Source: Open Mind (Camb). 2025 May 9;9:709–25. doi: 10.1162/opmi_a_00209 (PMC12140570; doi:10.1162/opmi_a_00209)
Supplement: Supplementary file 1 [file opmi-09-709-s001.pdf]

Supplemental Material for "Relative Value Encoding in Large Language Models: A Multi-Task, Multi-Model Investigation"

William M. Hayes   Nicolas Yax   Stefano Palminteri

1 Overview of Bandit Tasks

In each bandit task, the options were grouped into separate learning contexts, each containing a lower value option (L) and a higher value option (H). In the BP2023 task only, there were two ternary contexts containing a third, medium value option (M). Each option produced rewards from a Bernoulli (B2018, V2023, HW2023b) or Gaussian distribution (HW2023a, BP2023).

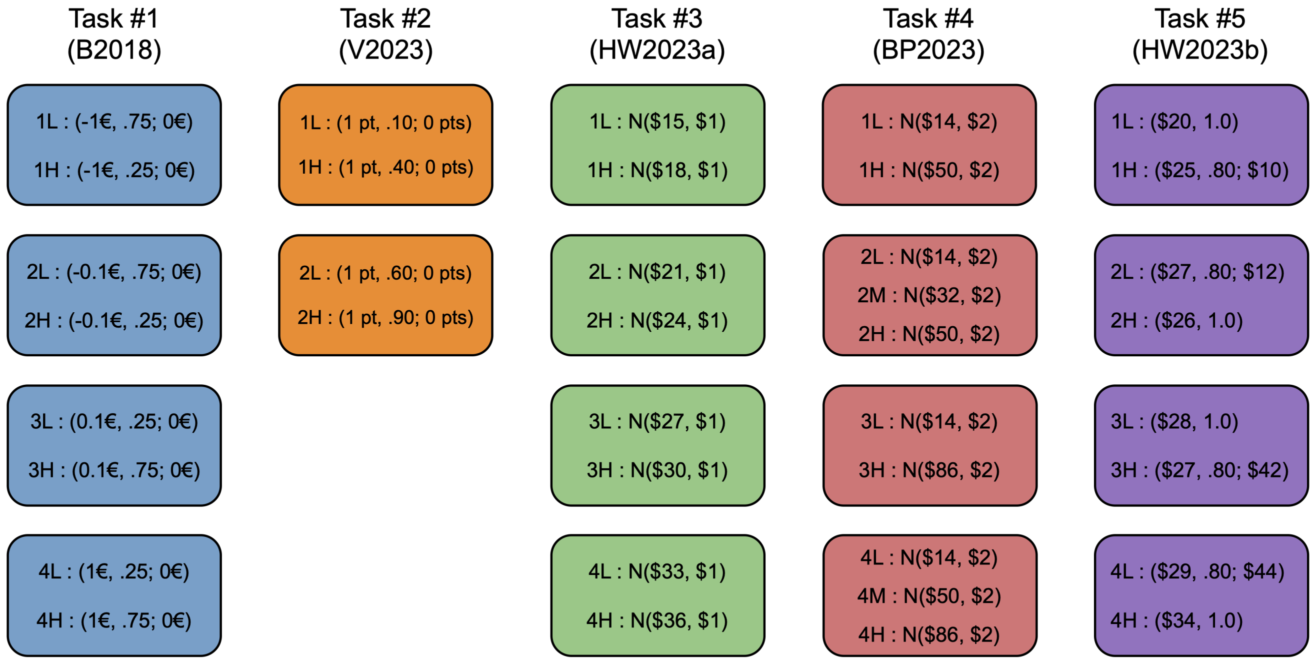

Figure S1: A list of the options in each bandit task. The notation $(x, p; y)$ means that x occurred with probability p, otherwise y. The notation $N(a, b)$ refers to a normal distribution with mean a and standard deviation b.

2 Task-Specific Prompts

Task #1 (B2018)

The aim of this task is to maximize your payoffs.
There are several slot machines that deliver wins and losses with different probabilities.
On each round, you will be asked which of two slot machines you wish to play.
Seeking monetary rewards and avoiding monetary losses are equally important.
Your total payoff will be the cumulative sum of the money you win across all rounds of the game.

Task #2 (V2023)

You are playing a game that involves choosing between different slot machines.
Each slot machine gives 1 point with a particular probability, otherwise 0 points.
Some slot machines have a higher probability of reward than others.
The goal is to maximize your total payoff over the course of several rounds.
Your total payoff will be the cumulative sum of the points you win across all rounds of the game.

Task #3 (HW2023a)

You are playing a game with the goal of winning as much money as possible over the course of several rounds.
In each round, you will be asked which of two slot machines you wish to play.
Some slot machines win more money than others on average.
Your total payoff will be the cumulative sum of the money you win across all rounds of the game.
Remember that your goal is to maximize your total payoff.

Task #4 (BP2023)

In this task, you will be given information about several slot machines in order to decide which ones you want to play.
Some slot machines win more money than others on average.
On each trial, you will be asked to choose between two or three different slot machines.
Your goal is to make choices that maximize your total payoffs. In other words, you should try to win as much money as possible.
Your total payoff will be the cumulative sum of the money you win across all rounds of the game.

Task #5 (HW2023b)

In this task, you will be given information about several slot machines in order to decide which ones you want to play.
Some slot machines win more money than others on average.
Your goal is to make choices that maximize your total payoffs. In other words, you should try to win as much money as possible.
Your total payoff will be the cumulative sum of the money you win across all rounds of the game.

Figure S2: Specific prompts used in each bandit task.

3 Choice Accuracy: Additional Results

Below are the results from the ANOVAs on choice accuracy (i.e., proportion of reward-maximizing choices) in the learning phase and transfer test. Primary focus was on the main effect of Prompt, which represents the average effect of prompt design across tasks and LLMs.

Table S1: ANOVA results for learning phase accuracy.

Source	*df*	SS	MS	F	p	η_p^2
Task	4	16.858	4.214	434.899	<.001	0.60
LLM	3	5.376	1.792	184.933	<.001	0.32
Prompt	1	1.428	1.428	147.391	<.001	0.11
Task × LLM	12	1.832	0.153	15.755	<.001	0.14
Task × Prompt	4	0.603	0.151	15.553	<.001	0.05
LLM × Prompt	3	1.222	0.407	42.039	<.001	0.10
Task × LLM × Prompt	12	0.582	0.048	5.004	<.001	0.05
Error	1160	11.241	0.010			

Table S2: ANOVA results for transfer test accuracy.

Source	*df*	SS	MS	F	p	η_p^2
Task	4	4.652	1.163	152.330	<.001	0.34
LLM	3	6.470	2.157	282.490	<.001	0.42
Prompt	1	0.409	0.409	53.516	<.001	0.04
Task × LLM	12	0.495	0.041	5.398	<.001	0.05
Task × Prompt	4	0.116	0.029	3.795	.005	0.01
LLM × Prompt	3	0.345	0.115	15.054	<.001	0.04
Task × LLM × Prompt	12	0.127	0.011	1.392	.163	0.01
Error	1160	8.856	0.008			

4 Relative Value Bias: Additional Results

Below are results from the analysis of transfer test trials where one option had a higher relative value than the other. An option's relative value was determined by the frequency with which it gave better relative outcomes than the other option(s) in the same learning context. For each task, we can compute the proportion of times an ideal, reward-maximizing agent would choose the option with higher relative value in the transfer test. If the empirical choice rate from an LLM exceeds the ideal choice rate, this is taken as an indicator of relative value bias.

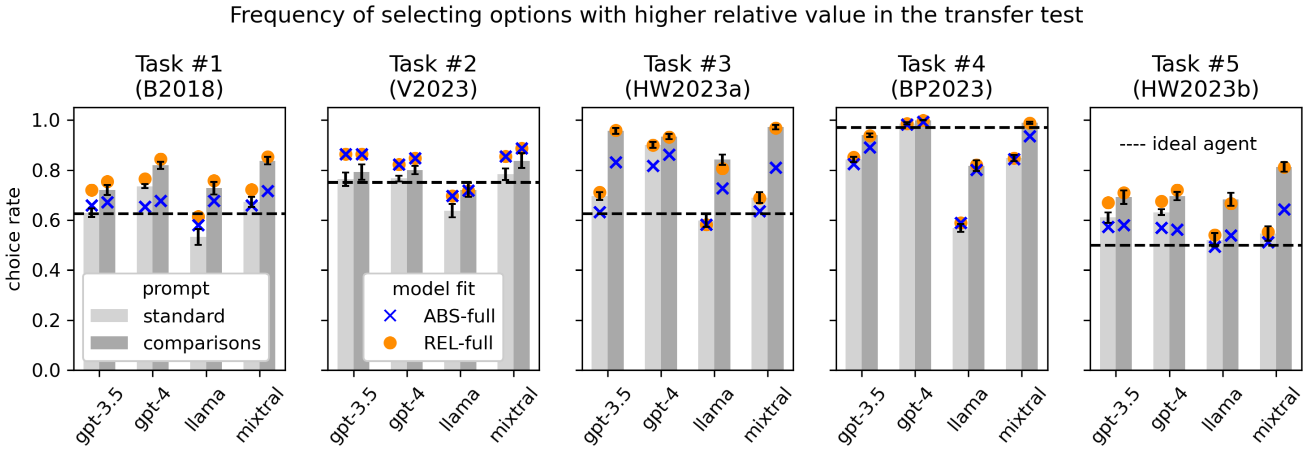

Figure S3: Choice rates for the options with higher relative value in the transfer test. Bars show the empirical data (+/- standard error). Dashed lines show the behavior of an ideal, reward-maximizing agent. The blue X symbols show the fit of the ABS-full model, which assumes unbiased outcome encoding. The orange O symbols show the fit of the REL-full model, which incorporates relative outcome encoding. Both models allow for separate learning rates and inverse temperature parameters. Though the ABS-full model is able to capture some of the relative value biases, the REL-full model fits the data better.

Table S3: ANOVA results for the choice rates of options with higher relative value.

Source	df	SS	MS	F	p	η_p^2
Task	4	7.60	1.90	162.14	<.001	0.36
LLM	3	4.48	1.49	127.56	<.001	0.25
Prompt	1	5.16	5.16	440.61	<.001	0.28
Task × LLM	12	1.26	0.11	8.87	<.001	0.08
Task × Prompt	4	0.73	0.18	15.61	<.001	0.05
LLM × Prompt	3	1.00	0.33	28.39	<.001	0.07
Task × LLM × Prompt	12	0.58	0.05	4.11	<.001	0.04
Error	1159	13.58	0.01			

Note: One observation was excluded due to missing data after removing trials with tied relative values.

5 Transfer Test Pairwise Choice Patterns

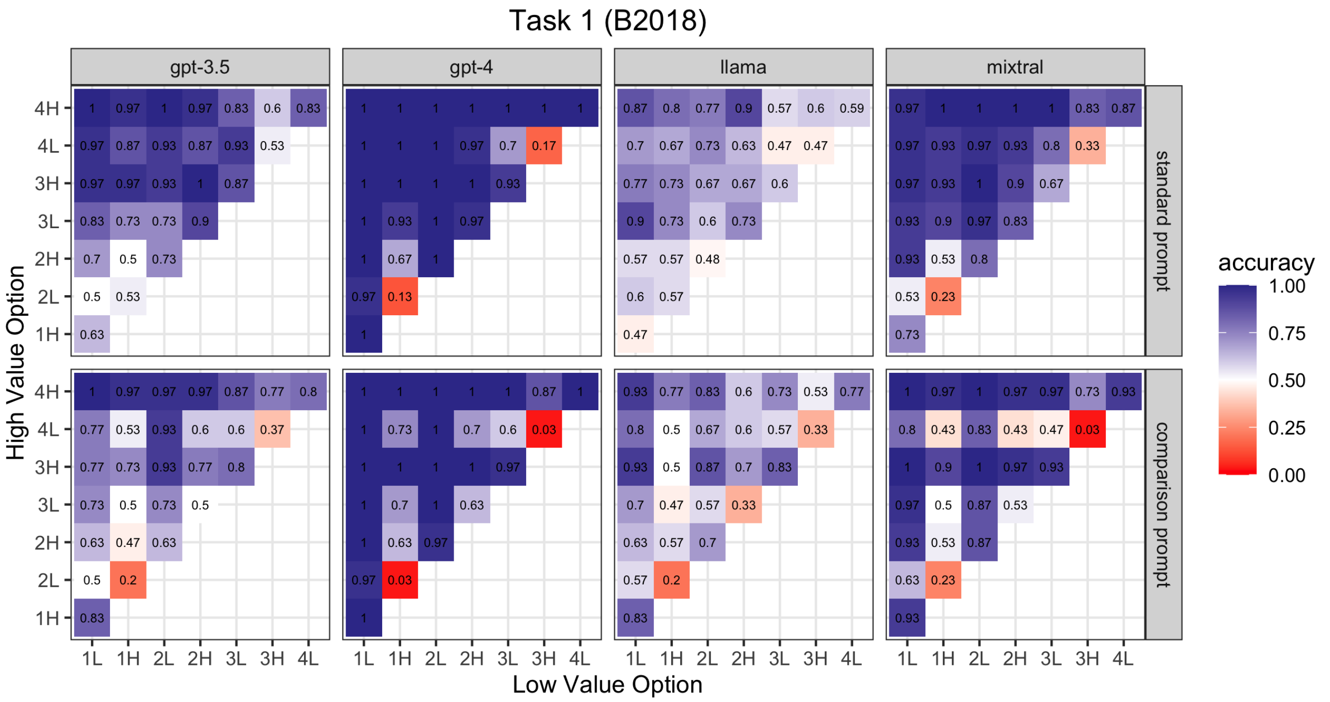

Figure S4: Each cell shows the proportion of runs in which the higher value option was chosen over the lower value option. Cells with accuracy < 0.5 (red) indicate violations of expected value maximization.

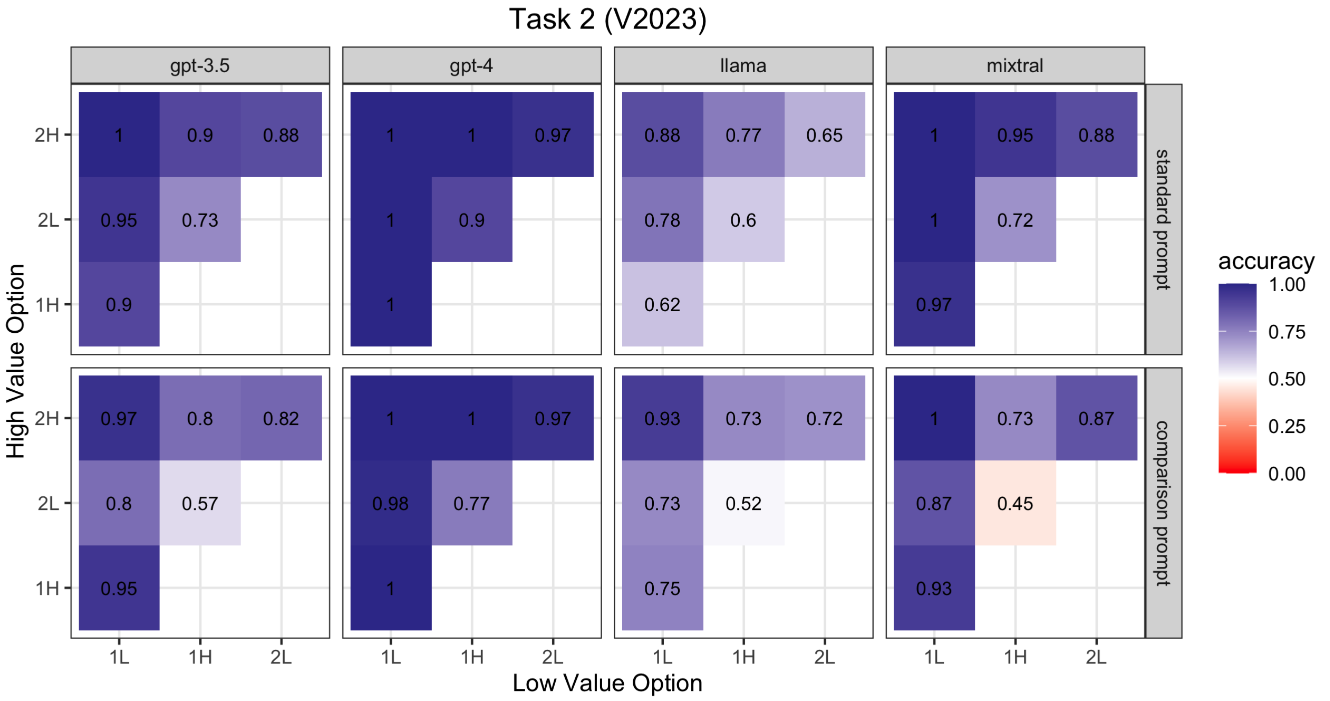

Figure S5: Each cell shows the proportion of runs in which the higher value option was chosen over the lower value option. Cells with accuracy < 0.5 (red) indicate violations of expected value maximization.

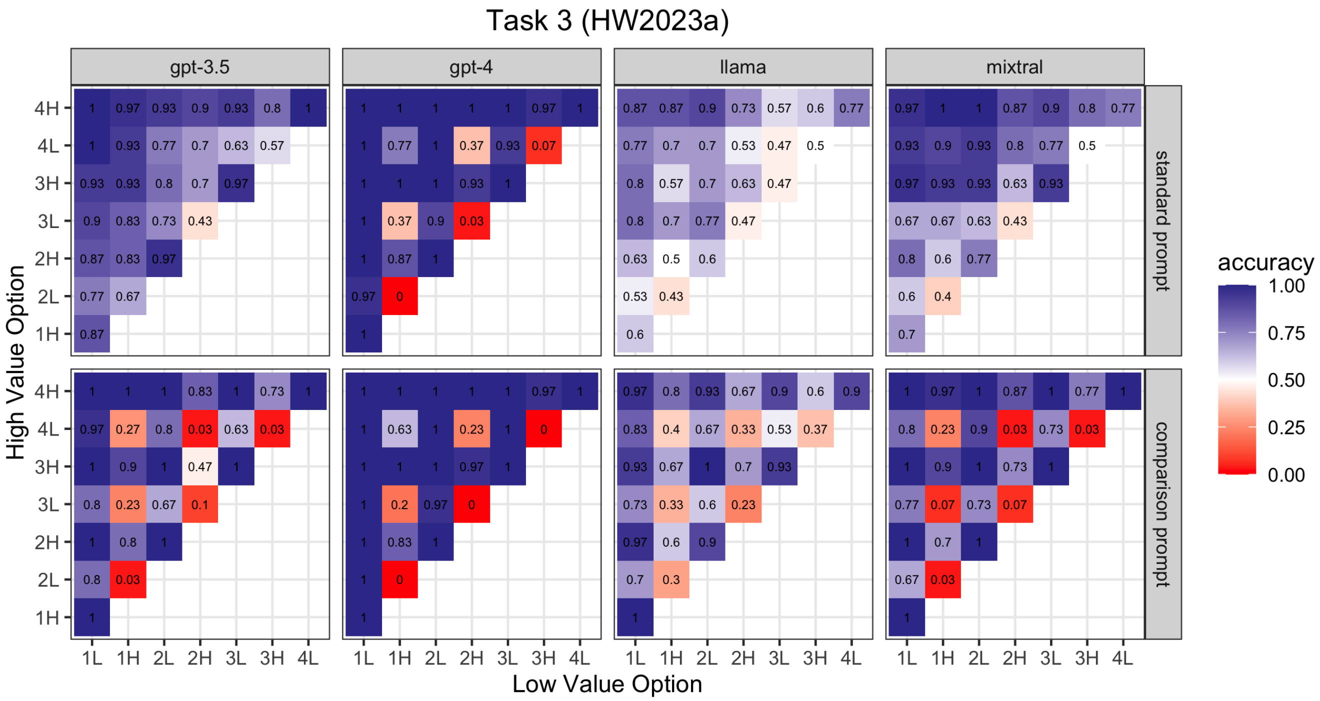

Figure S6: Each cell shows the proportion of runs in which the higher value option was chosen over the lower value option. Cells with accuracy < 0.5 (red) indicate violations of expected value maximization.

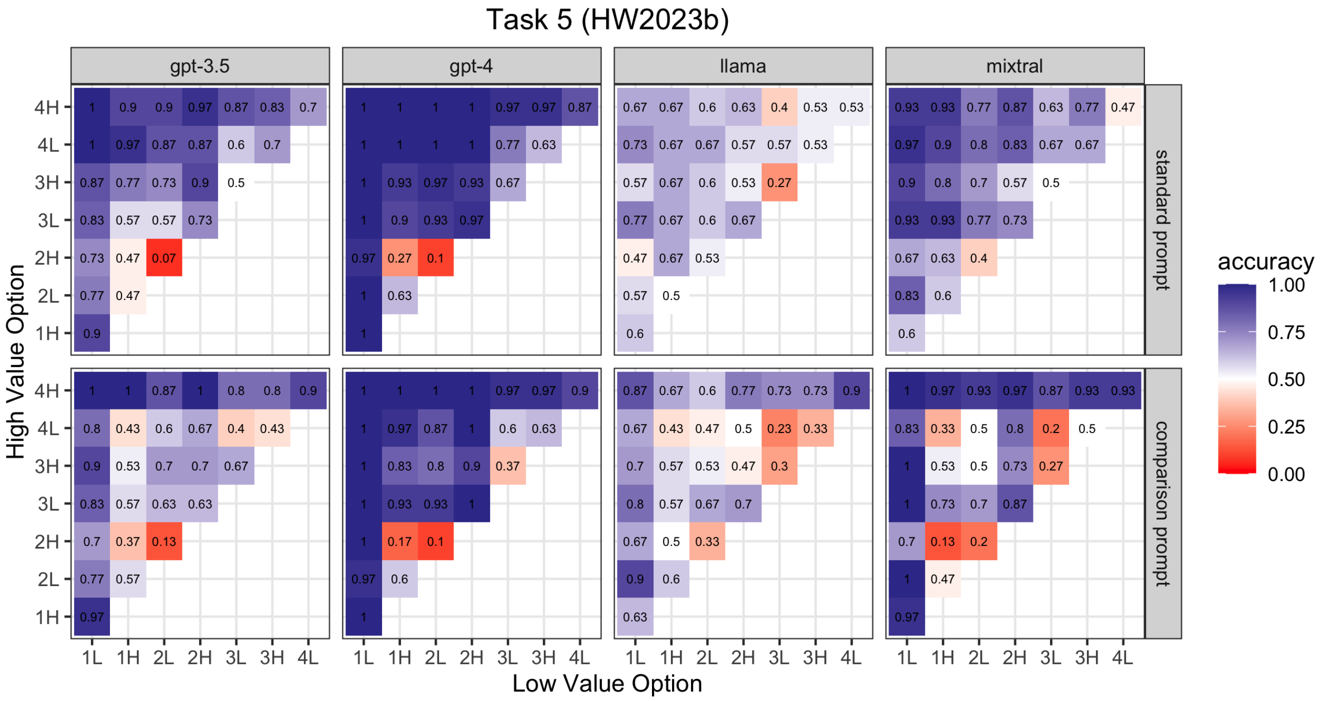

Figure S7: Each cell shows the proportion of runs in which the higher value option was chosen over the lower value option. Cells with accuracy < 0.5 (red) indicate violations of expected value maximization.

6 Model Recovery Simulations

Model recovery simulations were conducted to determine how distinguishable our cognitive RL models are across the five bandit tasks. For each task, 20 choice datasets were generated from each of the eight models (ABS, ABS-2α, ABS-2β, ABS-full, REL, REL-2α, REL-2β, REL-full). The data-generating parameters for each dataset were drawn from the following prior distributions: $\alpha \sim Uniform(0, 1)$, $\omega \sim Uniform(0, 1)$, $\beta \sim Uniform(0, 50)$, and $b \sim Uniform(-2, 2)$. We then fit each of the eight RL models to the generated data and selected the best-fitting model based on BIC values. If the models are distinguishable, the true data-generating model will be selected more frequently than the other models.

The confusion matrices in the top row of Figure S8 show, for each task, the conditional probability of selecting each of the eight candidate models, given the true data-generating model (columns sum to 1.0). When the true data-generating model had one learning rate (α) and one inverse temperature (β), it was successfully recovered 96% of the time on average. The more complex models with two learning rates and/or two inverse temperatures were recovered 56.5% of the time on average (chance = 12.5%). When the wrong model was selected, it was almost always a simpler model that possessed the correct outcome encoding mechanism (absolute or relative). The bottom row of Figure S8 shows the inverse conditional probability that each of the eight candidate models was the true data-generating model, given the selected model (rows sum to 1.0). When BIC values favored the more complex models, there was a high probability that the selected model was the one that generated the data (especially ABS-full or REL-full). The overall pattern of results suggests that the two outcome encoding mechanisms were highly distinguishable in these tasks, but less so in the V2023 task. Though recovery rates were above chance for all models, the simulations indicate a bias toward simpler models with fewer parameters.

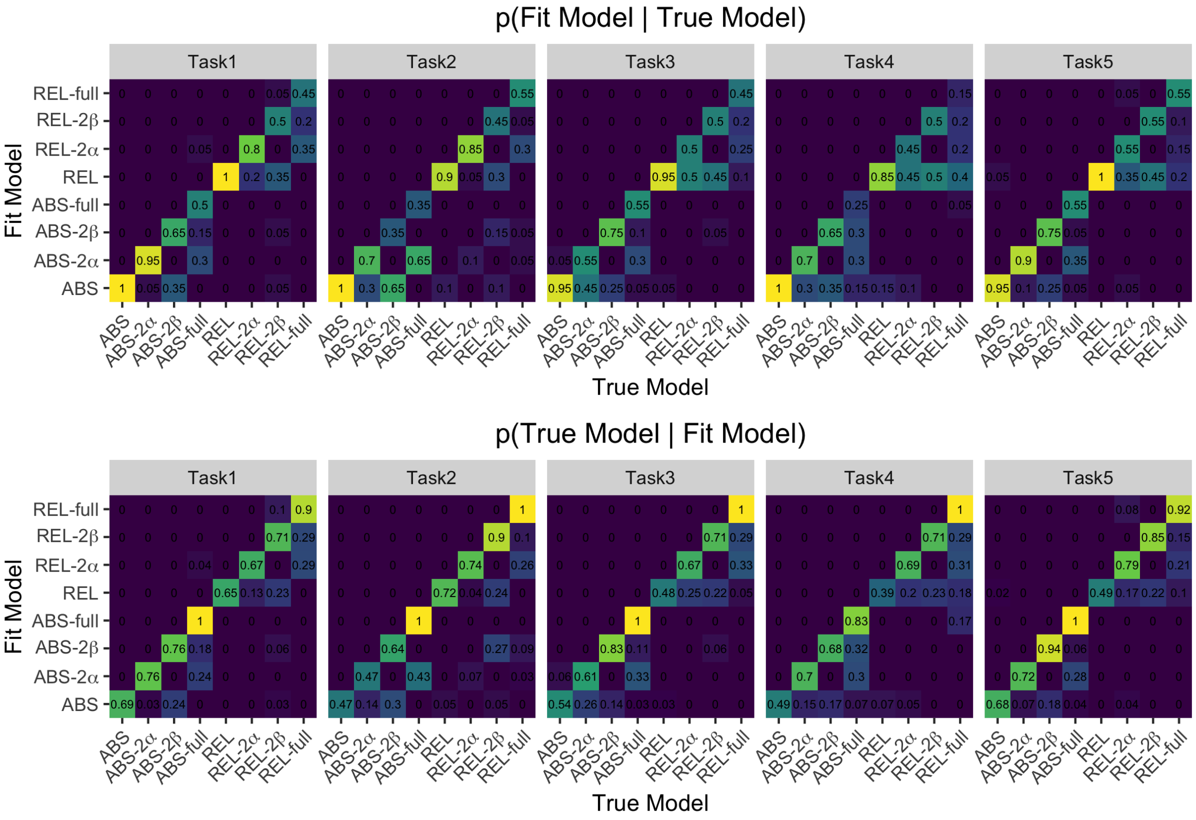

Figure S8: Model recovery results. Top: probability of each model being selected, given the true data-generating model (columns sum to 1.0). Bottom: probability of each model being the true data-generating model, given the selected model (rows sum to 1.0). Probabilities were computed across 20 simulations.

7 Model Comparisons

Table S4: Model comparison results based on Bayesian Information Criterion (BIC).

Task	Prompt	LLM	ABS	ABS-2α	ABS-2β	ABS-full	REL	REL-2α	REL-2β	REL-full
1	S	gpt-3.5	2124	2046	2131	2051	2090	2013	2096	2019
		gpt-4	1299	1182	1179	1163	972	978	950	958
		llama	2175	1976	2182	1982	2182	1959	2190	1962
		mixtral	2257	2227	2265	2233	2220	2199	2227	2204
	C	gpt-3.5	2289	1962	2243	1964	2114	1866	2113	1870
		gpt-4	1622	1409	1448	1398	1046	1051	1024	1031
		llama	2465	1944	2403	1946	2252	1831	2250	1838
		mixtral	2215	1977	2118	1983	1677	1638	1675	1639
2	S	gpt-3.5	1563	1319	1541	1319	1556	1327	1548	1327
		gpt-4	722	685	721	689	730	693	728	697
		llama	1836	1385	1843	1393	1844	1393	1852	1400
		mixtral	1501	1323	1503	1330	1498	1331	1504	1337
	C	gpt-3.5	2110	1588	2062	1587	2068	1596	2058	1595
		gpt-4	820	721	789	712	800	728	793	720
		llama	2036	1546	2015	1540	2010	1553	2015	1548
		mixtral	1637	1196	1540	1194	1523	1204	1514	1202
3	S	gpt-3.5	2060	1652	1533	1485	1532	1484	1456	1431
		gpt-4	1694	819	872	748	398	404	398	406
		llama	2518	2048	2470	2021	2453	2056	2457	2029
		mixtral	2503	2215	2441	2218	2360	2193	2366	2200
	C	gpt-3.5	2482	1144	1168	1021	653	648	604	613
		gpt-4	1813	734	911	696	323	322	314	318
		llama	2681	1738	2049	1637	1734	1571	1738	1552
		mixtral	2556	1426	1389	1198	730	727	730	730
4	S	gpt-3.5	1819	1818	1544	1550	1706	1714	1504	1512
		gpt-4	597	591	605	595	584	592	590	598
		llama	2715	2642	2915	2620	2722	2650	2703	2628
		mixtral	1911	1889	1913	1891	1914	1895	1918	1898
	C	gpt-3.5	1229	1213	1033	1034	1036	1043	942	950
		gpt-4	571	511	570	511	499	505	503	504
		llama	1658	1461	1595	1388	1485	1404	1457	1360
		mixtral	986	963	888	874	728	729	721	725
5	S	gpt-3.5	2719	2283	2629	2287	2395	2142	2329	2143
		gpt-4	2352	2057	2357	2035	1870	1799	1877	1796
		llama	2675	2277	2673	2261	2655	2192	2657	2189
		mixtral	3078	2647	3070	2638	3069	2641	3053	2627
	C	gpt-3.5	2885	2122	2589	2090	2423	1828	2186	1816
		gpt-4	2595	2279	2602	2273	1672	1667	1679	1675
		llama	2841	2339	2685	2311	2401	2069	2350	2060
		mixtral	3081	2547	3074	2553	2089	1981	2080	1987

Note: S = standard prompt, C = comparison prompt. BICs were rounded to the nearest integer for display purposes. The lowest BIC in each row is shown in red.

Table S5: Model comparison results based on cross-validated pseudo-R^2.

Task	Prompt	LLM	ABS	ABS-2α	ABS-2β	ABS-full	REL	REL-2α	REL-2β	REL-full
1	S	gpt-3.5	.332	.357	.331	.357	.343	.369	.343	.368
		gpt-4	.592	.629	.626	.631	.698	.698	.706	.705
		llama	.305	.371	.303	.371	.302	.377	.299	.377
		mixtral	.287	.298	.285	.297	.299	.307	.298	.307
	C	gpt-3.5	.278	.384	.294	.384	.335	.416	.337	.416
		gpt-4	.490	.559	.540	.560	.675	.674	.683	.682
		llama	.214	.384	.234	.385	.283	.418	.285	.416
		mixtral	.301	.378	.330	.373	.472	.486	.474	.487
2	S	gpt-3.5	.476	.560	.483	.562	.477	.560	.482	.561
		gpt-4	.759	.773	.761	.773	.758	.773	.761	.773
		llama	.377	.535	.374	.533	.376	.535	.376	.532
		mixtral	.494	.557	.494	.556	.495	.557	.494	.556
	C	gpt-3.5	.294	.471	.309	.473	.306	.471	.311	.473
		gpt-4	.728	.762	.739	.767	.733	.762	.738	.767
		llama	.308	.479	.315	.483	.316	.479	.316	.483
		mixtral	.452	.603	.483	.606	.488	.603	.493	.606
3	S	gpt-3.5	.441	.550	.585	.597	.587	.600	.608	.615
		gpt-4	.541	.781	.761	.804	.897	.897	.897	.896
		llama	.314	.446	.327	.451	.331	.444	.330	.451
		mixtral	.320	.399	.336	.399	.358	.406	.358	.405
	C	gpt-3.5	.327	.693	.687	.729	.827	.830	.843	.843
		gpt-4	.508	.805	.757	.813	.917	.919	.922	.922
		llama	.270	.530	.442	.550	.529	.574	.530	.582
		mixtral	.304	.616	.620	.679	.805	.804	.806	.806
4	S	gpt-3.5	.646	.648	.700	.700	.669	.669	.709	.708
		gpt-4	.886	.885	.885	.888	.888	.886	.886	.886
		llama	.366	.384	.368	.390	.364	.384	.364	.390
		mixtral	.627	.633	.628	.634	.627	.633	.627	.633
	C	gpt-3.5	.762	.758	.794	.789	.801	.801	.807	.805
		gpt-4	.890	.902	.891	.904	.895	.892	.896	.895
		llama	.582	.635	.598	.656	.627	.652	.635	.664
		mixtral	.809	.815	.826	.831	.861	.862	.863	.864
5	S	gpt-3.5	.259	.380	.285	.380	.348	.420	.367	.421
		gpt-4	.358	.441	.358	.449	.491	.512	.491	.514
		llama	.272	.383	.272	.388	.276	.405	.275	.408
		mixtral	.161	.280	.164	.283	.162	.282	.168	.287
	C	gpt-3.5	.213	.425	.297	.435	.338	.505	.401	.510
		gpt-4	.291	.378	.291	.382	.547	.549	.546	.549
		llama	.222	.361	.266	.366	.342	.436	.356	.440
		mixtral	.161	.307	.163	.306	.432	.464	.436	.464

Note: S = standard prompt, C = comparison prompt. Pseudo-R^2 was calculated as $1 - LL_M/LL_0$, were LL_M is the log-likelihood of the data under the model in question and LL_0 is the log-likelihood of the data under chance responding. The models were fit to 97% of the data and LL_M computed for the remaining 3% in a leave-one-out cross-validation procedure. The values in the table reflect averages across 30 cross-validation iterations. The highest pseudo-R^2 in each row is shown in red.

8 Task-Specific Choice Patterns and Model Fit

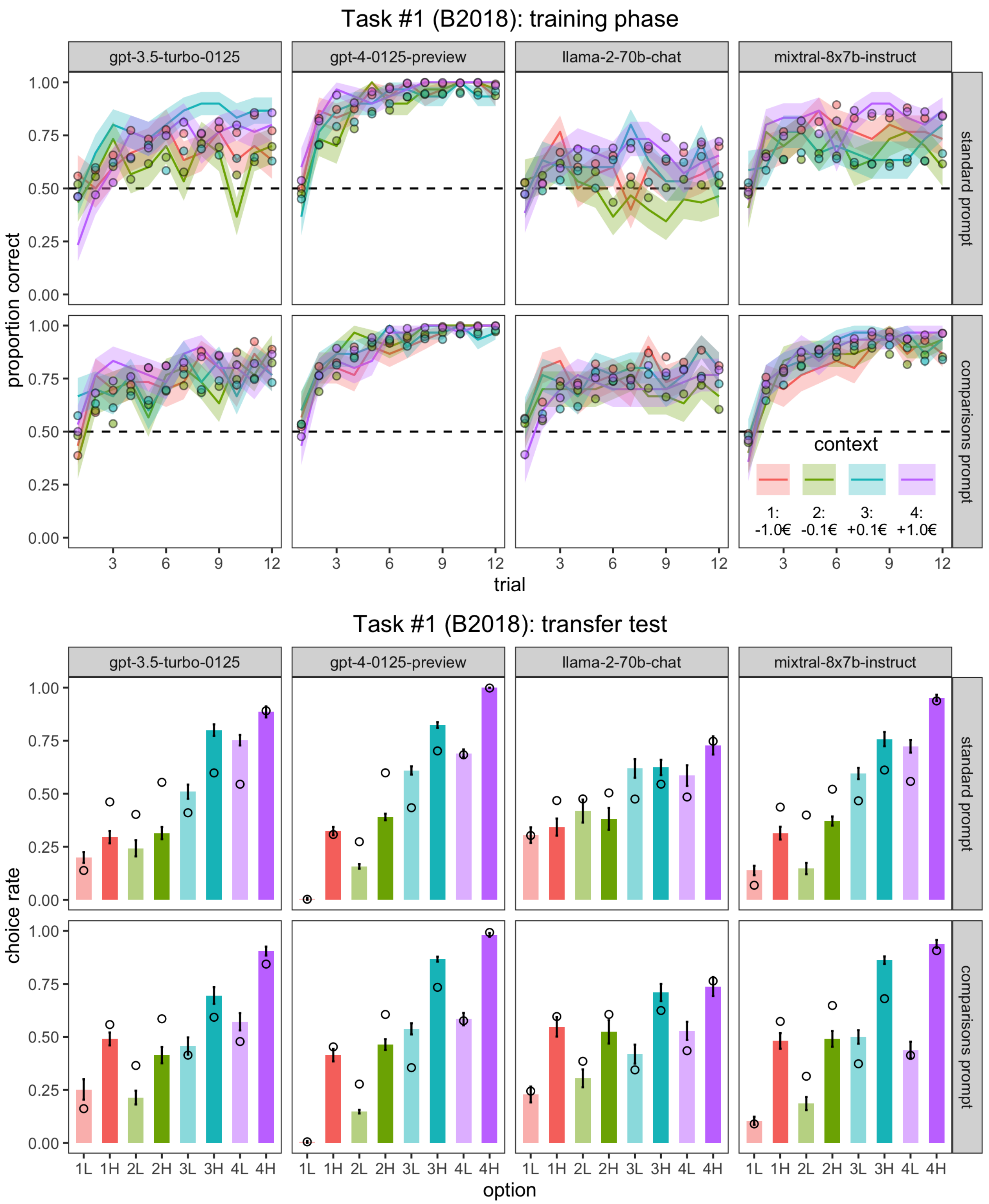

Figure S9: (a) Proportion of correct (reward-maximizing) choices across trials in the learning (training) phase. Lines show the empirical data (+/- 1 standard error). Points show the fit of the REL-full model. (b) Mean choice rates for each option in the transfer test. Bars show empirical data (+/- 1 standard error). Points show the fit of the REL-full model.

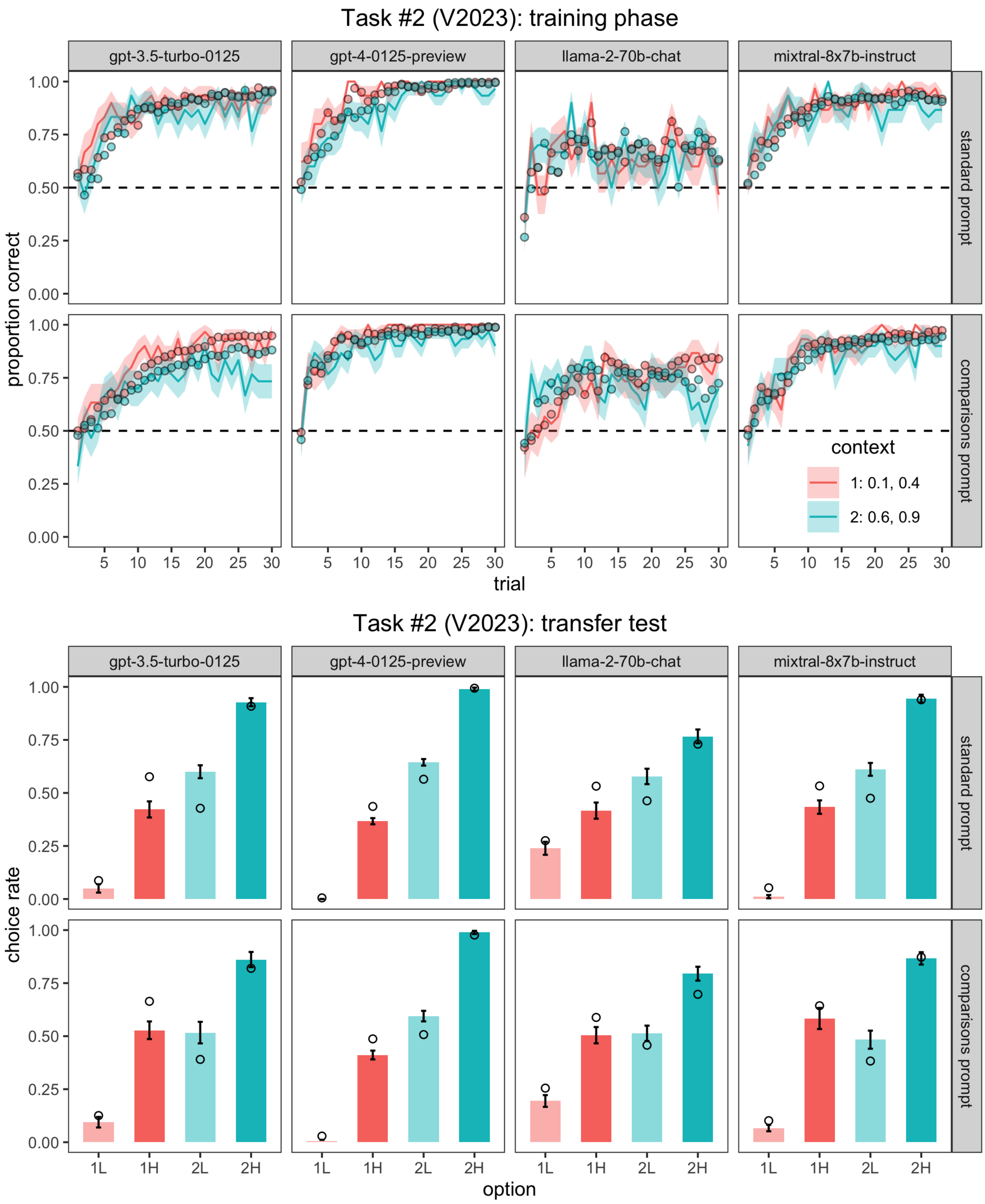

Figure S10: (a) Proportion of correct (reward-maximizing) choices across trials in the learning (training) phase. Lines show the empirical data (+/- 1 standard error). Points show the fit of the REL-full model. (b) Mean choice rates for each option in the transfer test. Bars show empirical data (+/- 1 standard error). Points show the fit of the REL-full model.

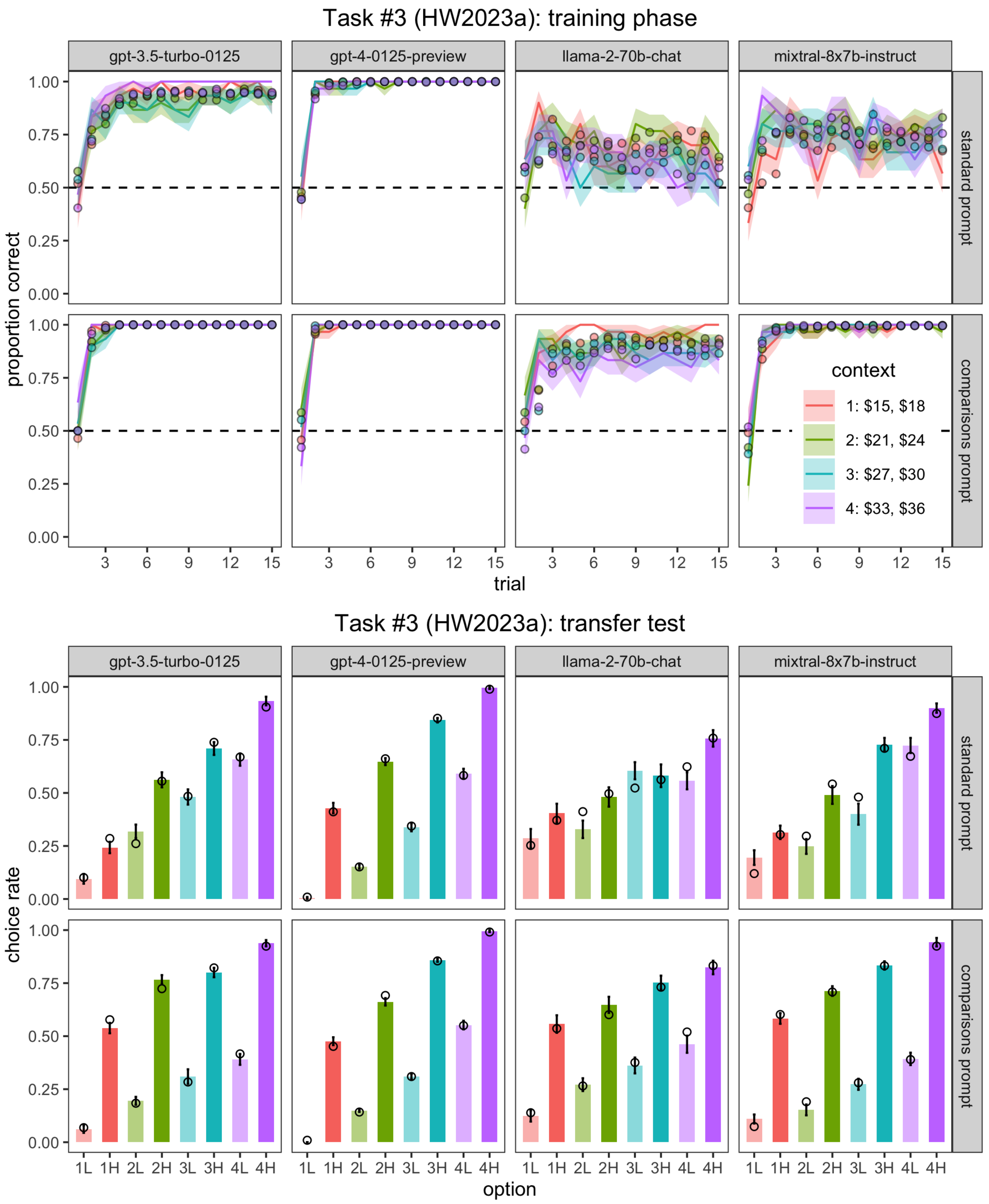

Figure S11: (a) Proportion of correct (reward-maximizing) choices across trials in the learning (training) phase. Lines show the empirical data (+/- 1 standard error). Points show the fit of the REL-full model. (b) Mean choice rates for each option in the transfer test. Bars show empirical data (+/- 1 standard error). Points show the fit of the REL-full model.

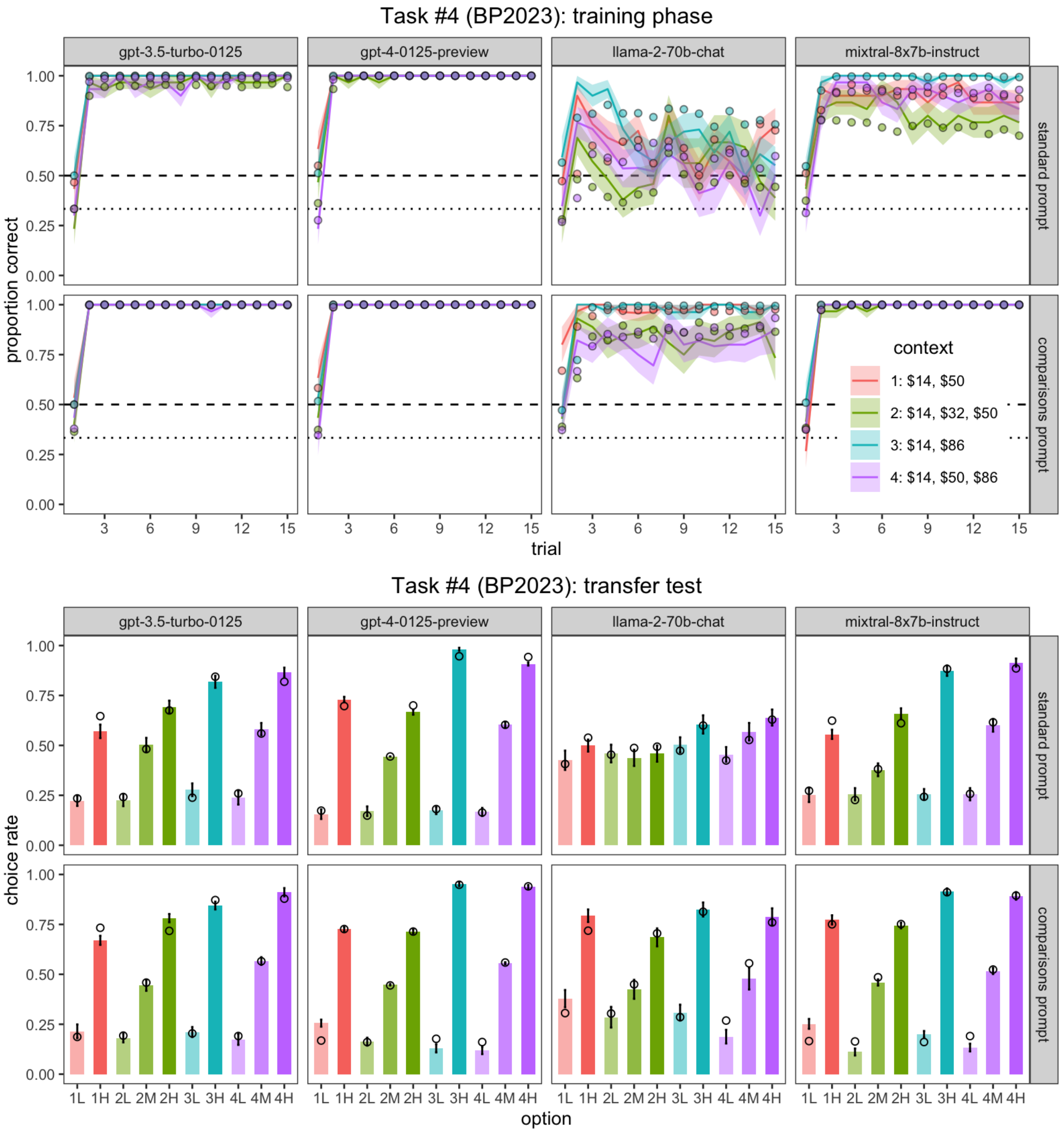

Figure S12: (a) Proportion of correct (reward-maximizing) choices across trials in the learning (training) phase. Lines show the empirical data (+/- 1 standard error). Points show the fit of the REL-full model. (b) Mean choice rates for each option in the transfer test. Bars show empirical data (+/- 1 standard error). Points show the fit of the REL-full model.

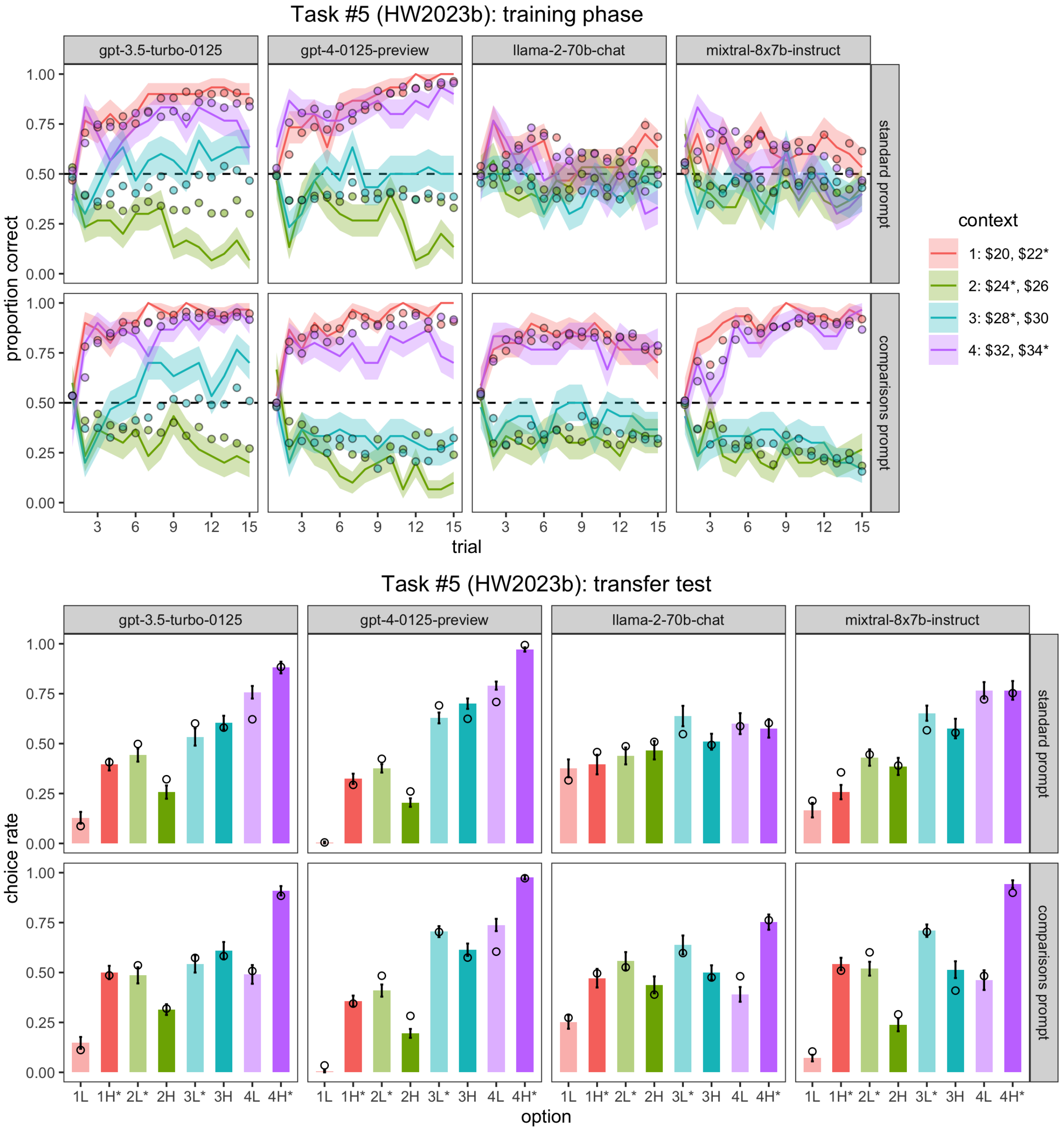

Figure S13: (a) Proportion of correct (reward-maximizing) choices across learning (training) phase. Lines show the empirical data (+/- 1 standard error). Points show the fit of the REL-full model. (b) Mean choice rates for each option in the transfer test. Bars show empirical data (+/- 1 standard error). Points show the fit of the REL-full model. In both plots, asterisks (*) designate the options that frequently gave better relative outcomes in their original training contexts.

9 Parameter Estimates

Below are the estimated parameters for the REL-full model across LLMs.

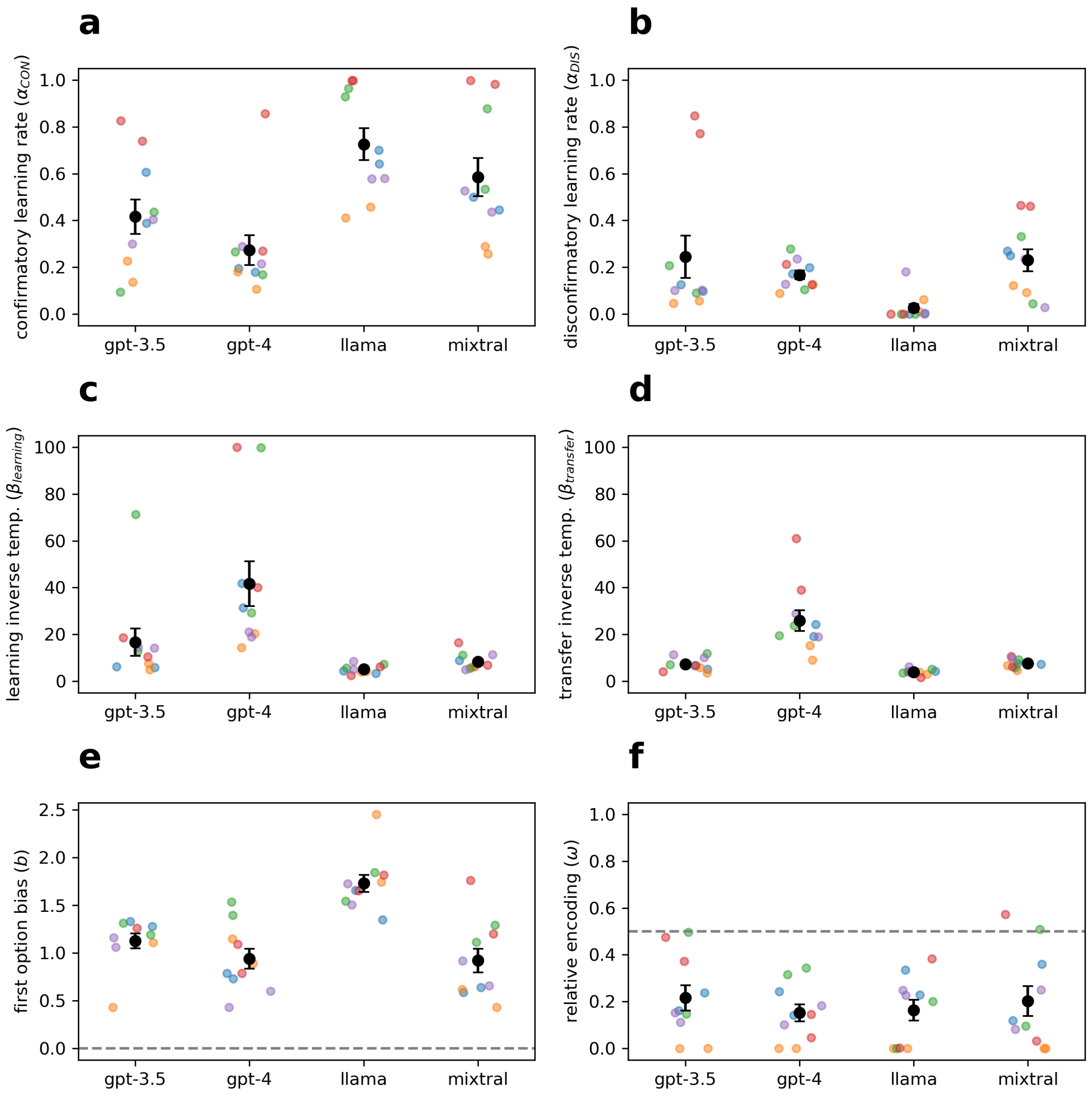

Figure S14: Parameter estimates from the REL-full model. (a-b) Learning rates. (c-d) Inverse temperatures. (e) First option bias. (f) Relative encoding. In each panel, the colored points show the estimates for a specific combination of task, prompt design, and LLM. The black points show the means and standard errors for each LLM.

10 Additional Analyses with gemma-7b

The following analyses were conducted using gemma-7b, a 7 billion parameter model developed by Google. We used the raw, pretrained version of the model available on Hugging Face. Gemma-7b was trained on 6T tokens of mostly English text data from web documents, mathematics, and computer code.

10.1 Relative value bias

Would gemma-7b, a pretrained model with no fine-tuning, exhibit a relative value bias? To test this, we ran gemma-7b through Task #3 (HW2023a) using a very similar procedure to what was used for the other models. Because gemma-7b was not fine-tuned for chat, the prompts were structured so that the model could simply complete the prompt with its choice. That is, the end of each prompt was as follows:

Prompt for gemma-7b

...
Q: Which slot machine do you choose?
A: I would choose slot machine [insert]

As shown in Figure S15, gemma-7b showed weak evidence of learning with no obvious signs of relative value bias using the standard prompt. When the comparison prompt was used, learning phase performance was considerably improved, but relative value biases were also clearly magnified in the transfer test.[1]

10.2 Analysis of hidden states

We asked whether the hidden layer activations in an LLM might encode task-relevant information, such as the difference in absolute or relative values between choice options on each trial. We had gemma-7b perform the transfer test in the HW2023a task, on each trial recording the final hidden layer activations for the last token in the prompt ("machine"). We repeated this procedure 100 times, resulting in a 2800 $\times$ 3072 matrix of activation values (2800 trials, 3072 hidden units). These experiments were run on a single cloud A100-80G GPU.

Then, for each choice trial, we computed the difference in the absolute values of the two choice options (first minus second), as well as the difference in the relative values, both normalized between 0 and 1. For example, if the choice were between options 1H and 3L (see Figure S1), the difference in absolute values would be $(18 - 27)/(36 - 15) = -0.4286$ and the difference in relative values would be $1.0 - 0.0 = 1.0$.

Linear regression was used to predict the trial-to-trial activations in each hidden unit from the trial-to-trial differences in absolute and relative values, plus an intercept. Each hidden unit was modeled separately, for a total of 3072 regressions. An extremely conservative significance threshold was used to account for the large number of tests: The significance of each of the 6144 slope coefficients (3072 regressions $\times$ 2 predictors) was tested using a critical p-value of $.001/6144 = 1.628 \times 10^{-7}$. The results are presented in the main text (see Figure 5).

[1]The results in Figure S15 were based on a version of the task with 60 learning trials. The results for the hidden states analysis described in section 10.2 were based a shorter version of the task with 40 learning trials. Other than the number of trials, the structure of the bandit task was the same in both sets of experiments.

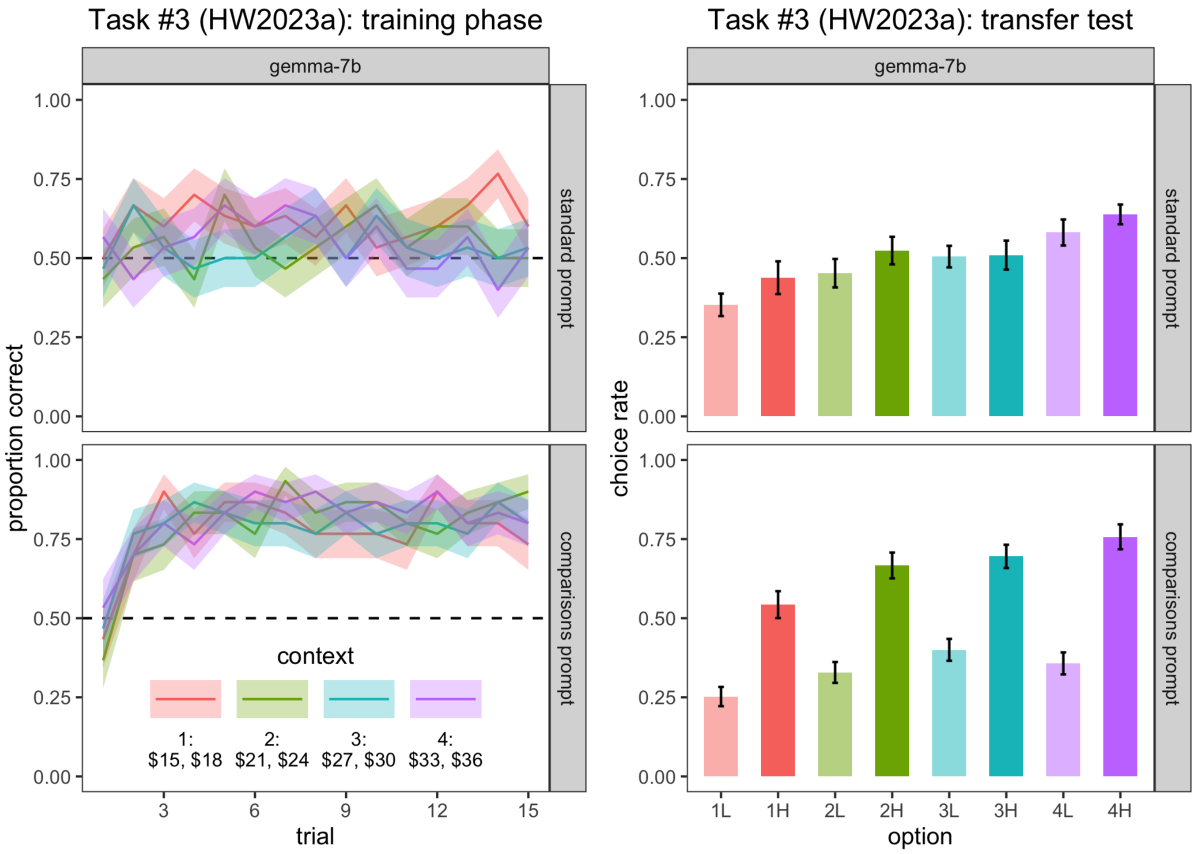

Figure S15: Experiments with gemma-7b. (left) Proportion of correct choices across learning (training) phase trials in both prompt conditions (+/- 1 standard error). (right) Mean choice rates for each option in the transfer test (+/- 1 standard error).
